# The XylS/*Pm* regulator/promoter system and its use in fundamental studies of bacterial gene expression, recombinant protein production and metabolic engineering

**DOI:** 10.1111/1751-7915.12701

**Published:** 2017-03-09

**Authors:** Agnieszka Gawin, Svein Valla, Trygve Brautaset

**Affiliations:** ^1^ Department of BiotechnologyNorwegian University of Science and TechnologyTrondheimNorway

## Abstract

The XylS/*Pm* regulator/promoter system originating from the *Pseudomonas putida* TOL plasmid pWW0 is widely used for regulated low‐ and high‐level recombinant expression of genes and gene clusters in *Escherichia coli* and other bacteria. Induction of this system can be graded by using different cheap benzoic acid derivatives, which enter cells by passive diffusion, operate in a dose‐dependent manner and are typically not metabolized by the host cells. Combinatorial mutagenesis and selection using the *bla* gene encoding β‐lactamase as a reporter have demonstrated that the *Pm* promoter, the DNA sequence corresponding to the 5′ untranslated end of its cognate mRNA and the *xylS* coding region can be modified and improved relative to various types of applications. By combining such mutant genetic elements, altered and extended expression profiles were achieved. Due to their unique properties, obtained systems serve as a genetic toolbox valuable for heterologous protein production and metabolic engineering, as well as for basic studies aiming at understanding fundamental parameters affecting bacterial gene expression. The approaches used to modify XylS/*Pm* should be adaptable for similar improvements also of other microbial expression systems. In this review, we summarize constructions, characteristics, refinements and applications of expression tools using the XylS/*Pm* system.

## Introduction

**Figure 1:**
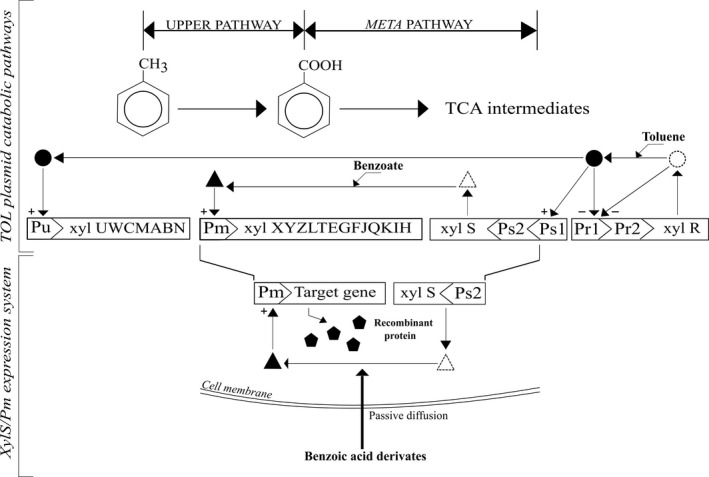
The upper scheme illustrates the catabolic operon for degradation of aromatic compounds in the *Pseudomonas putida* TOL plasmid pWW0. The upper‐pathway operon is under transcriptional control of XylR/*Pu* and encodes enzymes that transform toluene into benzoate. Subsequently, benzoate is converted to tricarboxylic acid (TCA) intermediates by enzymes encoded in the meta‐pathway operon, under transcriptional control of XylS/*Pm*. Circles, XylR; triangles, XylS; open symbols, regulator unable to activate transcription; solid symbols, regulator able to stimulate transcription; +, activating effect; −, repressing effect (Inouye *et al.,* 1987; Greated *et al.,* 2002). The lower part of the figure presents how XylS/*Pm* can be applied to express recombinant genes. *Pm* is activated by the XylS regulator when it forms a complex with a meta‐pathway substrate entering passively into the cell.

The *Pm* promoter and its cognate regulator gene *xylS* originate from the *Pseudomonas putida* TOL plasmid pWW0 and control expression of an operon encoding enzymes involved in the degradation of aromatic hydrocarbons (Worsey and Williams, 1975). The *xylS* gene encodes the AraC family positive transcriptional regulator XylS which upon binding to its effector becomes activated, binds as a dimer to its operator sequence and induces transcription from *Pm*. On pWW0, *xylS* is transcribed from two tandem promoters, *Ps1* and *Ps2. Ps1* is σ^54^‐dependent and inducible, while *Ps2* is σ^70^‐dependent and provides constitutive, low‐level expression of XylS (Gallegos *et al*., 1996) (Fig. 1). The XylS/*Pm* expression cassette including the *Ps2* promoter was initially used for the construction of broad‐host‐range vectors, which were based on the RSF1010 replicon and contain x*ylE* as a reporter protein. Regulated expression of XylE from vector pNM185 was demonstrated in 15 of totally 18 tested different Gram‐negative bacterial species (Mermod *et al*., 1986). Further modifications of pNM185 resulted in construction of plasmids pERD20 and pERD21 (Ramos *et al*., 1988) carrying a mutant *xylS* gene, designated *xylStr6*, with altered effector specificity. These latter plasmids were tested in *Escherichia coli* by using β‐galactosidase as a reporter gene and 3–8 fold elevated expression levels were obtained compared with the original vectors under *Pm* induction conditions. Moreover, pERD20 and pERD21 displayed a broad temperature range for inducible expression and functioned well in 7 different Gram‐negative bacterial species tested. These early studies showed for the first time that XylS/*Pm* has a potential for regulated recombinant expression in many different Gram‐negative species.

Later, the system was used in combination with the broad‐host‐range minimal replication elements *oriV* (origin of vegetative replication) and *trfA* (encodes the TrfA protein required for initiation of replication at *oriV* and also controls plasmid copy number) from the naturally occurring RK2 plasmid, resulting in a set of versatile plasmid vectors with known nucleotide sequences (Blatny *et al*., 1997a,b). In these vectors, the copy number is adjustable by introducing desired point mutations in the *trfA* gene. The vectors also contain polylinkers facilitating cloning of target genes at an ATG site appropriately positioned relative to the DNA sequence encoding the native *Pm* ribosome binding site to ensure efficient translation. A common feature for expression vectors containing XylS/*Pm* is that several alternative, non‐toxic and cheap benzoic acid derivatives can be used as inducers. They enter the cells by passive diffusion and function in a dose‐dependent manner, usually without being metabolized. These characteristics together with the properties of the expression cassette itself explain why XylS/*Pm* is such a useful tool for a variety of applications in many different bacterial species.

Despite the many favourable properties of wild‐type XylS/*Pm,* the system has been substantially improved by refinement of the *Pm* promoter sequence, the 5′‐untranslated region of *Pm*‐derived transcript, the *xylS* coding sequence and certain 5′‐terminal fusion partners. This allowed for a full exploitation of the potential of this expression cassette both for high‐level protein production and applications in which tightly controlled expression at more physiologically relevant levels is desired. The outcomes of such approaches have provided new genetic tools widening the range of further applications and have also contributed to new fundamental insights into bacterial gene expression. The methods used to achieve these results should be possible to apply for any expression cassette in principle in any host, although the technologies have so far been used almost exclusively in *E. coli*.

*E. coli* is among the most commonly used and best studied bacterial hosts for heterologous gene expression (for a review, see Rosano and Ceccarelli, 2014). Despite of this, functional heterologous protein production even in this host is still often a matter of trial and error, indicating that fundamental aspects of bacterial gene expression have not yet been fully understood. In this review, we describe many and, in several respects, unique properties of the XylS*/Pm* regulator/promoter system. We present its current applications in *E. coli* and many other bacterial species, most often for recombinant protein production and metabolic engineering types of experiments. Finally, we consider the potential of XylS/*Pm* as a valuable model system for basic studies on gene expression in bacteria.

## The architecture and functioning of the XylS/*Pm* expression cassette

**Figure 2:**
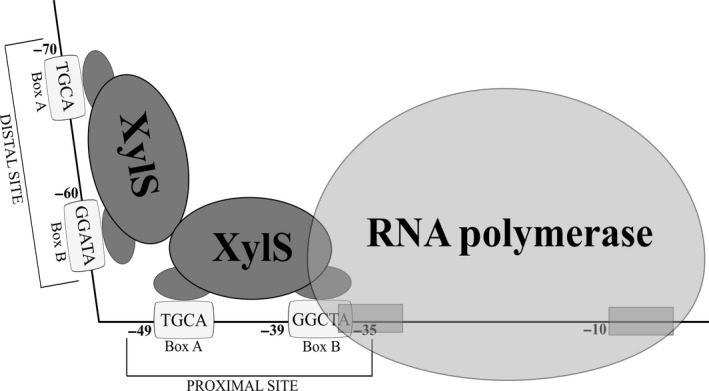
Schematic model of *Pm* activation. XylS binding sites and the ‐10/‐35 consensus sequences for binding RNA polymerase are indicated. As a result of the interactions between XylS monomers and the *Pm* region, the DNA curvature increases to an overall of 98° (González‐Pérez *et al.,* 1999, 2002; Domínguez‐Cuevas *et al.,* 2010).

XylS is a positively regulating transcription factor belonging to the AraC‐XylS family, and when activated by a benzoate‐derived inducer, it binds to an operator sequence and initiates transcription from the *Pm* promoter (Fig. 2). The inducer molecules can appear in protonated or non‐protonated states depending on the growth medium pH. Only the protonated forms can diffuse passively into the cells, meaning that the expression level when using this system may also be affected by pH (Winther‐Larsen *et al*., 2000a,b). Transcription initiation from *Pm* is mediated by the XylS/inducer complex and σ^32^‐dependent RNA polymerase in early exponential growth phase or σ^38^‐dependent RNA polymerase in early stationary phase and thereafter (Marqués *et al*., 1999; Domínguez‐Cuevas *et al*., 2005).

The origin and biology of the AraC‐XylS family of transcription activators and their regulation have been extensively reviewed previously (Gallegos *et al*., 1997; Ramos *et al*., 1997; Martin and Rosner, 2001; Egan, 2002; Tobes and Ramos, 2002; Schleif, 2003 Brautaset *et al*., 2009; Santiago *et al*., 2016) and will therefore be only briefly outlined here. XylS is a 321 amino acid protein with a molecular mass of 36 kDa (Domínguez‐Cuevas *et al*., 2008). To date, no experimental 3D structure of XylS exists as this protein is, like many AraC members, poorly soluble which so far has rendered its purification in active form unsuccessful (Aune *et al*., 2010; Domínguez‐Cuevas *et al*., 2010). XylS appears to be composed of two separate and functionally independent domains: a conserved *C*‐terminal domain (CTD) for DNA binding and interactions with RNA polymerase and an *N*‐terminal domain (NTD) responsible for effector recognition and protein dimerization (Gallegos *et al*., 1997). Double mutants harbouring two specific single amino acid substitutions in the *N*‐terminal domain of XylS have been constructed (Ruíz and Ramos, 2001, 2002), and the mutant protein displayed altered induction properties confirming the role of this domain for the effector binding. It has also been demonstrated that the *C*‐terminal domain alone is able to activate transcription as efficiently as the full‐length protein (Kaldalu *et al*., 2000), while the *N*‐terminal domain represses DNA binding in the absence of benzoate effectors (Domínguez‐Cuevas *et al*., 2010). XylS‐CTD consists of seven α‐helices folding into two helix–turn–helix (HTH) motifs. Two recognition helices (α‐helix 3, α‐helix 6) are critical for establishing contact with the *Pm* promoter in a region organized as two homologous 15‐base pairs direct repeats, each consisting of a 5′‐box A (TGCA) and a 3′‐box B (GGTA) separated by 6 base pairs (Fig. 2). Recognition of these direct repeat sequences by XylS leads to the formation of a dimer acting in two consecutive steps. The first XylS monomer occupies the proximal binding site and facilitates binding of the second monomer to the distal site. Binding of the two monomers provokes a gradual bending of DNA and interaction of XylS with RNA polymerase as illustrated in Fig. 2 (Kessler *et al*., 1993; González‐Pérez *et al*., 1999, 2002; Domínguez‐Cuevas *et al*., 2008, 2010). Activation of XylS may be caused either by effector binding or by XylS hyper‐production leading to auto‐induction independent of any inducer. One striking feature is that a high number of different benzoic acid‐based compounds function as inducers for XylS (Silva‐Rocha *et al*., 2011). To date, 59 different compounds have been tested and among these 31 compounds were reported to induce expression from *Pm* with different induction ratios (Table 1).

**Table 1:** Reported XylS effectors and their induction ratios

Inducer	Induction ratios^a^	Selected references
3‐methylbenzoate (m‐toluate)	17–600	Mermod *et al*. (1986), Ramos *et al*. (1990a); Zhou *et al*. (1990), Michan *et al*. (1992a), Blatny *et al*. (1997a), Winther‐Larsen *et al*. (2000b), Cebolla *et al*. (2002), Ruíz *et al*. (2003), Aune *et al*. (2010), Zwick *et al*. (2013)
Salicylate	15–292^b^	Ramos *et al*. (1990a), Michan *et al*. (1992a), Cebolla *et al*. (2001, 2002), Royo *et al*. (2005a, b), Medina *et al*. (2011), Mesa‐Pereira *et al*. (2013, Mesa‐Pereira *et al*. (2015) )
Acetyl salicylic acid (ASA)^b^	20–150	Royo *et al*. (2007)
3‐methylsalicylate^b^	46–72	Ramos *et al*. (1988), Cebolla *et al*. (2002)
5‐methoxysalicylate^b^	46	Cebolla *et al*. (2002)
Benzoate^b^	44	Ramos *et al*. (1990a); Zhou *et al*. (1990), Cebolla *et al*. (2001, 2002), Jiménez *et al*. (2014), Silva‐Rocha and de Lorenzo (2014)
2‐methylbenzoate	18–29^b^	Ramos *et al*. (1990a); Zhou *et al*. (1990), Michan *et al*. (1992a), Frerichs‐Deeken *et al*. (2003), Purvanov and Fetzner (2005)
4‐chlorobenzoate	5–26^b^	Ramos *et al*. (1990a), Nielsen *et al*. (2000)
4‐ methoxybenzoate	1–23^b^	Ramos *et al*. (1990a), Michan *et al*. (1992a), de Lorenzo *et al*. (1993)
3‐chlorobenzoate	11–22^b^	Ramos *et al*. (1990a), Nielsen *et al*. (2000), Liu *et al*. (2010)
4‐methylbenzoate	4–21^b^	Ramos *et al*. (1990a); Zhou *et al*. (1990), Michan *et al*. (1992a)
5‐methylsalicylate^b^	19	Cebolla *et al*. (2002)
2‐chlorobenzoate	14–18^b^	Ramos *et al*. (1990a)
2,3‐dimethylbenzoate	10–18^b^	Ramos *et al*. (1990a)
2‐methoxybenzoate	1–17^b^	Ramos *et al*. (1990a), Cebolla *et al*. (2002)
3‐fluorobenzoate	8–16^b^	Ramos *et al*. (1990a)
2‐fluorobenzoate	4–15^b^	Ramos *et al*. (1990a)
4‐ethylbenzoate	1–15^b^	Ramos *et al*. (1990a)
3‐bromobenzoate	12–14^b^	Ramos *et al*. (1990a)
4‐fluorobenzoate	4–14^b^	Ramos *et al*. (1990a)
4‐bromobenzoate	1–14^b^	Ramos *et al*. (1990a)
2,5‐dimethylbenzoate	1–13^b^	Ramos *et al*. (1990a); Zhou *et al*. (1990), Michan *et al*. (1992a)
3,4‐dichlorobenzoate	4–12^b^	Ramos *et al*. (1990a), Michan *et al*. (1992a)
anthranilate^b^	11	Cebolla *et al*. (2002)
2‐acetylsalicylate^b^	10	Cebolla *et al*. (2002)
3‐iodobenzoate	5–9^b^	Ramos *et al*. (1990a)
4‐iodobenzoate	1–9^b^	Ramos *et al*. (1990a)
3‐ methoxybenzoate	1–8^b^	Ramos *et al*. (1990a)
3,4‐dimethylbenzoate	5–7^b^	Ramos *et al*. (1990a)
2‐bromobenzoate	1–7^b^	Ramos *et al*. (1990a)
4‐methylsalicylate^b^	7	Cebolla *et al*. (2002)

The following compounds promoted no induction of the XylS/*Pm* or the induction ratios were not reported: 2,3‐dichlorobenzoate (Liu *et al*., 2010), 3,5‐dichlorobenzoate (Ramos *et al*., 1990a; Michan *et al*., 1992a; Liu *et al*., 2010), sodium benzoate (Purvanov and Fetzner, 2005), 5‐chlorosalicylate, 4‐chlorosalicylate, 3,5‐dichlorosalicylate (Cebolla *et al*., 2002), 2,4‐dimethylbenzoate (Ramos *et al*., 1990a; Zhou *et al*., 1990; Michan *et al*., 1992a), 2,6‐dichlorobenzoate (Ramos *et al*., 1990a; Michan *et al*., 1992a), 3,5‐dimethylbenzoate (Ramos *et al*., 1990a; Zhou *et al*., 1990), 2,6‐difluorobenzoate, 2‐iodobenzoate, 2,4‐dichlorobenzoate, 3‐hydroxybenzoate, 4‐hydroxybenzoate, 2,5‐dichlorobenzoate (Ramos *et al*., 1990a), 2,6‐dimethylbenzoate (Zhou *et al*., 1990), m‐xylene, o‐chlorotoluene, p‐ethyltoluene, 1,2,3‐trimethylbenzene, 1,3,4‐trimethylbenzene, 2,5‐dichlorotoluene, 2,6‐dichlorotoluene, benzyl alcohol, p‐methylbenzyl alcohol, p‐ethylbenzyl alcohol, m‐chlorobenzyl alcohol (Abril *et al*., 1989).

**a.** The ratio of the induced/basal expression. Induction was performed by using inducers at a concentration between 0, 1 and 5 mM (in most cases 1 or 2 mM).

**b.** The activity of indicated inducer or presented induction ratio was reported for the mutagenized form of XylS protein.

## Combinatorial mutagenesis and selection to alter and improve the elements of the XylS/*Pm* expression system

**Figure 3:**
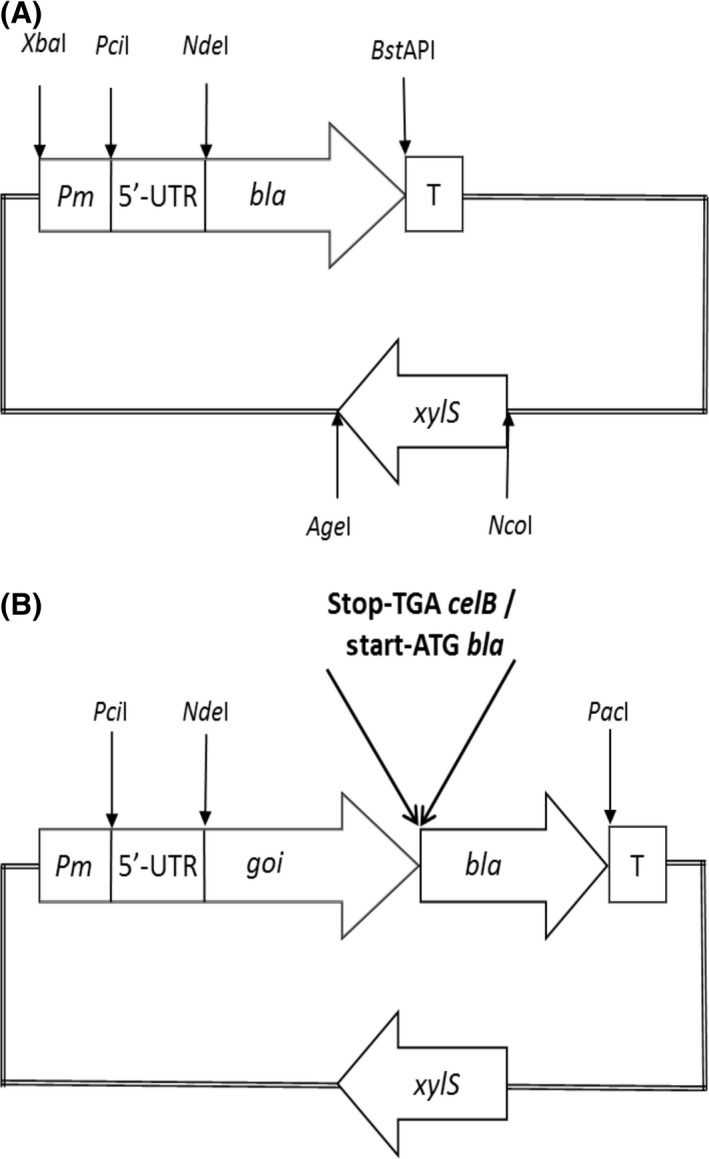
Vectors for combinatorial mutagenesis of various expression elements using *bla* as reporter gene for antibiotic tolerance level selection. A. Vector tool for mutagenesis and selection using *bla* (encoding β‐lactamase) as a reporter gene. The *Pm* promoter coding region, the *Pm* 5′‐UTR coding region and the *xylS* coding region can individually be substituted by libraries of randomly mutagenized oligonucleotides and genes. The libraries were made by synthesizing one mutated strand for each of the three regions. During synthesis of these strands, the three alternative nucleotides were mixed at a varying percentage (for example, 4% each) with the nucleotide of the wild‐type strand. The bases to be mutagenized were varied in different libraries. After synthesis, the DNA strands were annealed to their respective non‐mutagenized complementary strands (Winther‐Larsen *et al*., 2000b; Bakke *et al*., 2009). Cloning was then done by using the relevant restriction endonuclease sites indicated on the figure. Up and down mutants can be directly selected by growing the recombinant cells on different ampicillin concentrations. T is a transcriptional terminator. B. Vector tool for selection of 5′‐UTR mutants based on translational re‐initiation. The gene of interest (*goi*) is placed under control of *Pm* and the *bla* coding gene with its translational start codon overlaps with the *goi* stop codon (TGATG). Construction of mutant libraries of the *Pm* 5′‐UTR coding region and screening for increased ampicillin resistance relative to wild‐type was done as described in A above.

Directed evolution combining random mutagenesis protocols and selection has turned out to be more efficient than rational site‐directed mutagenesis to improve enzymes, indicating that our current understanding of the correlation between protein structure and function is still limited. The same is, at least to some extent, true also for gene expression elements. Despite the substantial knowledge accumulated about DNA sequences and features of promoters, spacer regions, ribosome binding sites and 5′‐untranslated mRNA regions, rational engineering of new and better expression systems remains challenging and highly unpredictable. Separate elements of the XylS/*Pm* system, i.e. the *Pm* promoter, the DNA sequence corresponding to the 5′‐untranslated mRNA region (5′‐UTR) of the *Pm‐*derived transcript, the *xylS* coding region, as well as various types of 5′‐terminal fusion partners that can stimulate expression of recombinant genes, have been modified and improved by using random mutagenesis and screening approaches. The common procedure has been to generate huge mutant libraries of the relevant genetic elements (i.e. by using doped oligonucleotides, error‐prone PCR, DNA shuffling, or combinations thereof). Such elements were further cloned into plasmid vectors harbouring the *bla* gene (encodes β‐lactamase), put under control of *Pm* and transformed to *E. coli*. Mutants were then directly selected by plating the heterogeneous recombinant cultures on solid medium containing different ampicillin concentrations, taking advantage of the fact that host ampicillin tolerance level correlates with the expression level of the *bla* gene (Winther‐Larsen *et al*., 2000a,b). The main focus has been on elevated expression levels, but the technology can be used to identify many other types of mutants too. The typical design and features of the selection vectors are illustrated in Fig. 3. These efforts resulted in construction of new and better expression tools expanding the properties and application range of XylS/*Pm*, and the experiments have also provided new basic understanding of genetic parameters affecting bacterial gene expression. Below, we summarize the most important findings from these experiments.

### Generation of Pm promoter and 5′‐UTR mutants displaying higher expression levels

Plasmid pJT19bla has the *bla* gene under transcriptional and translational control of *Pm* and *Pm‐*derived transcript including its *rbs* respectively (Winther‐Larsen *et al*., 2000b). In this vector, the region upstream of the *Pm* transcriptional start site and the region corresponding to the 5′‐UTR of the mRNA could be replaced in one‐step cloning procedures by doped oligonucleotides carrying random nucleotide substitutions relative to the wild‐type sequence (see Fig. 3A). By using this method, the desired regions could be mutagenized to an extent predetermined by the manner of synthesis of the oligonucleotides. Libraries of plasmids representing different *Pm* promoter and 5′‐UTR mutants were constructed in *E. coli* and variants leading to altered expression properties were selected by plating the recombinant cells on solid media containing different ampicillin concentrations. Point mutations (in most cases several in each clone) leading to enhancements of induced expression levels and mutations causing reduced background expression were identified. Combination of such mutations in some cases resulted in generation of expression cassettes with strongly extended induction windows compared with the wild‐type system. *Pm* promoter mutants with very high basal expression were also identified, indicating that background expression from both wild‐type and altered *Pm* promoter sequences is independent of XylS (Winther‐Larsen *et al*., 2000b). Later, these studies were extended by mutagenizing a larger region of the *Pm* promoter and by expansion of the mutant library (Bakke *et al*., 2009). First *Pm* mutants with up to 10‐fold elevated expression level of the *bla* reporter gene were selected, and the best mutant was then used as a template for a second round of random mutagenesis and selection. In this way, *Pm* promoter mutants with up to 14‐fold elevated expression level of reporter genes were eventually selected. Interestingly, mapping of the mutations causing the improved phenotypes revealed that the DNA sequence alternations were apparently randomly distributed in the mutagenized region in a presumably unpredictable manner (Bakke *et al*., 2009).

The 5′‐UTR of *Pm*‐derived transcripts contains the *rbs* consisting of the Shine–Dalgarno (SD) motif, the AUG initiation codon and a spacer region separating them, as well as upstream nucleotides. Initially, site‐directed mutagenesis was used to generate 12 different mutants with 1–6 nucleotide substitutions in the DNA sequence corresponding to the *rbs* of *Pm*‐derived transcripts (denoted as ‘SD mutants’) and the effects of these mutations on expression from *Pm* were tested by using the phosphoglucomutase gene *celB* as a reporter (Brautaset *et al*., 1998, 2000). The obtained results demonstrated that the SD mutations caused 1.5‐ to 50‐fold reduced expression level of CelB in *E. coli*. By using two alternative reporter genes, it was also shown that the effects of these SD mutants were highly gene specific (Winther‐Larsen *et al*., 2000b). The experiments were later extended by constructing a 5′‐UTR mutant library consisting of more than 25 000 variants and by selecting for up and down mutants using *bla* as a reporter gene, as described above (Berg *et al*., 2009). A number of 5′‐UTR mutants, all carrying mutations located outside of the SD sequence and causing up to 20‐fold elevated expression of β‐lactamase were in this manner selected. This indicated that the native SD region is likely already close to optimal for high‐level expression from *Pm*. Surprisingly, quantitative PCR analyses revealed that these 5′‐UTR mutations caused up to 7‐fold elevated *bla* transcript levels in the cells. By using alternative reporter genes, 5′‐UTR mutants causing up to 15‐fold increased transcript level from *Pm* were eventually isolated (Berg *et al*., 2009). For one selected 5′‐UTR mutant, it was deduced that this effect was not caused by increased mRNA stability or any alternation within the *Pm* transcription start site. This was the first documentation that the 5′‐UTR coding sequence can have a high impact on the transcription level of the cognate gene in *E. coli*.

By using *celB* as an alternative reporter gene, it was observed that the effects of the 5′‐UTR mutations were context dependent. Therefore, the vector system was redesigned to enable selection of 5′‐UTR mutants optimal for efficient expression of any recombinant gene (Berg *et al*., 2012). This time, *celB* and *bla* were cloned as a synthetic operon under control of *Pm* and with an overlap between the *celB* translational stop codon and the translational start codon of the *bla* gene. No *rbs* associated with the *bla* start codon was included, and any translation was thus dependent on translational reinitiation by ribosomes translating the upstream *celB* coding sequence of the mRNA (see Fig. 3B). In this way, any 5′‐UTR mutations causing increased transcription and/or translation of *celB* should directly cause elevated β‐lactamase production levels eventually affecting the ampicillin tolerance level of the recombinant cells. By using this novel selection tool, 5′‐UTR mutants causing up to 3‐fold elevated *celB* transcription level and 1.5‐fold elevated CelB production level were selected (Brautaset *et al*., 1998, 2000), demonstrating that this dual selection approach indeed functioned. Thus, because of its flexibility, the XylS/*Pm* cassette represents a valuable model system for basic studies aiming at expanding our understanding of genetic features affecting gene expression in bacteria.

### Mutagenesis of the xylS coding region and selection of mutants with altered functional properties

As described above, XylS has a non‐conserved *N*‐terminal domain for effector binding and protein dimerization and a conserved *C*‐terminal domain important for DNA binding and interactions with the host RNA polymerase. For identification of the specific regions involved in effector binding and determination of the models for XylS‐mediated *Pm* activation, a large number of *xylS* mutations resulting in different amino acid substitutions within *C*‐ and *N*‐terminal ends of XylS were constructed. The isolated XylS mutant proteins exhibited constitutively mediated transcription from *Pm* (Zhou *et al*., 1990), increased basal *Pm* activity and altered effector specificity and affinity (Ramos *et al*., 1990a,b; Michan *et al*., 1992a,b; Kessler *et al*., 1994). It was shown that XylS and its mutants can bind and respond differentially to many different chemical inducer molecules (Table 1) and when overproduced in the cells XylS can activate transcription from *Pm* in the absence of any inducer. Interestingly, amino acid substitution Phe291‐Tyr in the second helix–turn–helix motif of XylS resulted in a mutant with a significantly higher activity than wild‐type XylS (Manzanera *et al*., 2000).

Aune *et al*. (2010) used a combination of error‐prone PCR and DNA shuffling to randomly mutagenize the *xylS* coding region. XylS mutants with new effector profiles were then selected by using the *bla* reporter gene under the control of *Pm* (see above). Initially, a library of 430 000 *xylS* mutants was constructed in *E. coli* and screened for increased ampicillin tolerance level under induction conditions. The 40 most promising mutants contained totally 14 different amino acid substitutions within the *xylS* coding region and presented up to 3‐fold stimulation of expression from *Pm* compared with wild‐type XylS. Interestingly, all identified *xylS* mutations were located in the region encoding the *N*‐terminal domain of XylS. Combination of two or more of the selected mutations in one *xylS* gene generated unpredictable results. Therefore, 28 of the best mutant *xylS* genes were used as templates for DNA shuffling to recombine random combinations of mutations, and then the resulting library was exposed to a second round of screening for high levels of expression from *Pm*. This approach identified *xylS* variants with several beneficial mutations causing up to 10‐fold increased ampicillin host tolerance under induction conditions. One of these *xylS* mutant genes (StEP‐13) used together with the wild‐type *Pm* promoter allowed for 9‐fold increased protein production level of a single‐chain antibody variable fragment denoted scFv‐phOx (Sletta *et al*., 2004) in comparison with the wild‐type *xylS* (Aune *et al*., 2010). However, it was later shown that at high inducer and XylS concentrations the wild‐type and the StEP‐13 proteins resulted in similar maximum expression levels (Zwick *et al*., 2013).

### 5′‐terminal DNA sequences that encode protein translocation signal sequences can strongly affect the recombinant gene expression level from Pm

5′‐terminally fused DNA sequences encoding signal peptides for Sec pathway dependent protein translocation might confer an unexpectedly high impact on the expression level of heterologous proteins in *E. coli* (Sletta *et al*., 2007), and the choice of an optimal signal sequence is presumably protein dependent in an unpredictable manner (Li, 2015). A novel model system based on XylS*/Pm* was designed, enabling selection of improved signal sequences affecting the expression level and/or the translocation efficiency of recombinant proteins in *E. coli*. More specifically, a plasmid vector (pCSP1bla) was constructed in which the *bla* gene, with its native signal sequence, was replaced with a synthetic signal peptide denoted CSP (Sletta *et al*., 2004) and put under control of *Pm* (Heggeset *et al*., 2013). This approach takes advantage of the fact that β‐lactamase must be both expressed and translocated into periplasm to confer its biological function, and any mutations in the CSP coding region that could improve expression and/or translocation of this protein should result in elevated ampicillin tolerance of the host cells. The CSP coding region was randomly mutagenized by using doped oligonucleotides and the resulting library consisting of ca 137 000 clones was screened for mutants with increased ampicillin tolerance level. In this way, CSP mutants causing up to 5.5‐fold elevated expression and translocation of β‐lactamase were identified. Bioinformatics‐based analyses of around 20 different selected CSP variant DNA sequences could not rationally explain the obtained results. Interestingly, some of the CSP mutants could be also used for efficient expression and translocation of several alternative heterologous proteins in *E. coli*. These results highlighted the importance of optimizing the 5′‐terminal region of coding genes for their efficient expression in *E. coli* and also demonstrated that rational approaches using available bioinformatics tools could apparently not be applied to predict the outcomes. To decouple any potential effects of translocation process itself, the 5′‐terminal region of the *celB* gene (Brautaset *et al*., 1994) was tested as an alternative fusion partner to overexpress the human interferon alpha 2b (IFN‐α2b) gene intracellularly (Kucharova *et al*., 2013). *celB* can be functionally expressed to very high levels, while IFN‐α2b is poorly expressed, using XylS/*Pm* in *E. coli* (Brautaset *et al*., 1998, 2000; Winther‐Larsen *et al*., 2000b; Bakke *et al*., 2009; Berg *et al*., 2012). Totally 13 different *celB* fusion partners of varying lengths were fused in frame with the 5′‐end of the IFN‐α2b coding sequence, and expressed under control of the *Pm* promoter. The *celB* fusion partners ranging from 24 to 207 nucleotides long caused between 7‐fold and 60‐fold stimulation of expression at the transcript and protein levels, respectively, in *E. coli*. Further mutagenesis of the selected *celB* fusion sequences allowed for additional improvements which were also shown to be useful for high‐level heterologous production of IFN‐α2b in *E. coli* under high‐cell density cultivations (Kucharova *et al*., 2013) (see below).

### Combining optimized mutant genetic elements to expand the expression window of XylS*/Pm*

Overall, by using combinatorial engineering approaches, several different genetic elements of the XylS/*Pm* expression systems including the *Pm* promoter, the DNA sequence corresponding to its mRNA 5′‐UTR, the *xylS* coding region as well as external fusion partners (*celB*‐derived regions and the CSP translocation signal sequence) have been modified to improve both transcription, translation and translocation of heterologous proteins in *E. coli*. Zwick *et al*. (2012) reported that the β‐lactamase expression level could be up to 75‐fold and 50‐fold increased at the protein and transcript levels, respectively, by combining optimized *Pm*, 5′‐UTR and *xylS* regions. Similar results were obtained when using alternative reporter proteins. It was shown that even a single copy of such a multisite modified XylS/*Pm* expression cassette integrated into the *E. coli* chromosome could confer higher recombinant β‐lactamase production level than the analogous wild‐type plasmid present in multiple copies per genome (Zwick *et al*., 2013).

## Comparison of XylS/*Pm* performance with other relevant expression systems for regulated and high‐level heterologous gene expression in *E. coli*

Several different expression systems have been developed and work well for heterologous protein production in *E. coli* (Terpe, 2006; Brautaset *et al*., 2009; Tegel *et al*., 2011). For example, the T7 promoter originating from bacteriophage T7 (Studier and Moffatt, 1986) is recognized by its strength associated with the affinity of the highly selective T7 RNA polymerase which provides effective transcription initiation and *in vitro* elongation rate of 250 nucleotides per second compared with 50 for *E. coli* RNA polymerase (Golomb and Chamberlin, 1974). Placing the T7 polymerase gene under control of the *Plac* promoter allows induction of transcription from the *PT7* promoter by adding isopropyl‐β‐D‐1‐thiogalactopyranoside (IPTG) as an inducer. This system is, in contrast to XylS/*Pm,* based on negative transcriptional regulation mediated by the LacI repressor. A similar control mechanism is applied in case of the strong synthetic *Ptrc* promoter, which has been used to express heterologous proteins up to 15–30% of total cell protein in *E. coli* (Terpe, 2006). Another popular expression system is AraC*/P*_*BAD*_ in which the positive regulator AraC stimulates transcription from *P*_*BAD*_ upon induction with arabinose. Balzer *et al*. (2013) made an extensive comparative analysis of LacI/*PT7lac*, LacI/*Ptrc*, AraC/*P*_*BAD*_, wild‐type XylS/*Pm* and its high‐level expression variant *Pm* ML1‐17, in *E. coli* hosts. The main premise of the study was standardization of the design of the vectors to reduce influence of parameters unrelated to the features of the expression systems themselves. The most apparent observation following from these experiments was that the LacI/*PT7lac* system generates uniquely high amounts of transcripts. This property, however, typically correlates with an overload of the translational machinery eventually resulting in production of insoluble and inactive proteins. When considering protein functionality, weaker promoters sometimes allow to obtain higher yields of soluble and correctly folded proteins. In terms of low background expression, LacI/*Ptrc* turned out to be most leaky and it also displayed the smallest induction window. The most tightly regulated promoter system was AraC/*P*_*BAD*_. Calculation of translation initiation rate (TIR) values indicated that AraC/*P*_*BAD*_ and LacI/*PT7lac* transcripts are theoretically characterized by the most efficient translation. LacI/*PT7lac* and XylS/*Pm* ML1‐17 tended to produce the highest amount of recombinant proteins while XylS/*Pm* ML1‐17 showed higher yields of active proteins per transcript. One general advantage of using XylS/*Pm* is that it does not require any host‐mediated inducer uptake system, and most often the inducer is not consumed. In contrast, the *PT7* promoter needs host strains expressing the T7 RNA polymerase, usually from the *Plac* promoter. In case of AraC/*P*_*BAD*_, bacterial hosts should preferably be unable to catabolize the L‐arabinose inducer, but must be able to take up this compound. The XylS/*Pm* system is therefore easy to adapt to new bacterial hosts, what makes making it a very attractive candidate when the conditions of recombinant protein production have not previously been standardized.

Recently, a comparative microfluidic single‐cell analysis of LacI/*PT7lac*, AraC/*P*_*BAD*_ and XylS/*Pm* ML1‐17 in the synthetic M9CA growth medium was reported (Binder *et al*., 2016). Such well‐defined experimental set‐up provided high environmental homogeneity. The focus was preliminary on investigating the influence of different inducer molecules including their concentrations, and uptake mechanisms, on phenotypic heterogeneity as well as other system specifications. It was demonstrated that IPTG induction of LacI/*PT7lac* analysed in the *E. coli* strain BL21 (DE3) with an active lactose uptake mechanism, and salicylate induction of XylS/*Pm* ML1‐17 analysed in *E. coli* strain Tuner (DE3), led to the strongest initial expression and significant growth impairment. In contrast, analogous induction with IPTG using *E. coli* Tuner (DE3) with a passive lactose uptake mechanism and m‐toluate induction of XylS/*Pm* ML1‐17 showed intermediate responsiveness and hardly any interference with growth compared with respective non‐inducing conditions. Analysis of leaky expression confirmed the results obtained by Balzer *et al*. (2013) (see above) indicating that AraC/*P*_*BAD*_ is the most tightly regulated among the expression systems tested. Interestingly, XylS/*Pm* induced either by m‐toluate or salicylate revealed significant leaky expression leading to the subsequent moderate dynamic ranges of induction. Authors concluded that observed high basal expression of the system was probably triggered by use of mutagenized high‐level expression variant of *Pm* promoter (ML1‐17). Finally, expression responses of XylS/*Pm* induced by m‐toluate and LacI/*PT7lac* induced by IPTG in the absence of the active lactose uptake mechanism were characterized as the most homogenous. Summarized, it was proven that the type of inducer and the presence of inducer uptake systems can have a high impact on phenotypic heterogeneity, and this should be considered when choosing between different promoter systems.

In a separate study (Royo *et al*., 2005a), the performance of different promoter systems for expression of dioxygenase genes in *E. coli* was investigated. Comparison of the rate of indigo accumulation in the recombinant strains revealed that the induction level of *Pm* was slightly higher than in case of the *PT7* and *Ptac* promoters. However, all of these multicopy plasmid‐based systems were unstable when serially diluted batch experiments were performed without a selective pressure. The problem was solved by integrating the *Pm* expression module into the bacterial chromosome. Despite the gene dosage reduction and initially slower accumulation rate, the chromosomal system allowed for tightly controlled and stable production of indigo in amounts comparable to a multicopy plasmid, or a different plasmid system based on the *tac* promoter. In general, expression systems based on strong promoters like *Ptac*,* Ptrc* and *PT7* are rather considered to be unstable and very little is known about their performance after single copy integration into the chromosome (Royo *et al*., 2005a).

Sometimes utility of the promoter system may be limited by its host specificity. Bi *et al*. (2013) demonstrated that among all tested promoter systems, *PBAD* and *Pm* provided the highest expression level of the red fluorescent protein (RFP) upon induction in *Ralstonia eutropha*, whereas the *PlacUV5*,* Ptet* and *Ppro* systems were not functional in this host. These results argue in favour of the broad‐host‐range properties of the XylS/*Pm* regulatory cassette.

## Application of XylS/*Pm* for heterologous protein production under high‐cell density cultivations in *E. coli*

In addition to the laboratory‐scale experiments aiming at high‐level protein production, the XylS/*Pm* expression system has also been tested under more industrially relevant conditions. The expression system was reported to be useful for high‐level production of different human medical proteins, including granulocyte–macrophage colony‐stimulating factor (GM‐CSF), IFN‐α2b and a single‐chain antibody variable fragment (scFv‐phOx) as well as recombinant fish vaccines under high‐cell density cultivations (HCDC) of *E. coli* (Sletta *et al*., 2004, 2007; Tøndervik *et al*., 2013). Under such conditions, low background expression and strong induction have proven to be critical to obtain a high‐level of cell growth in the fermenter prior to *Pm* induction, leading to high volumetric production yields and preventing unwarranted loss of plasmids from the recombinant cells during the growth phase. Single‐chain antibody fragment scFv‐phOx must be translocated to the periplasm to fold into soluble and functional form. It is also regarded as host toxic in the sense that its overexpression and translocation eventually cause lysis of the recombinant cells. Thus, careful regulation of scFv‐phOx expression is critical in particular under HCDC. In agreement with what was described above, it was demonstrated that fusion sequences optimized by combinatorial mutagenesis and selection could stimulate high‐level expression also of other heterologous protein in *E. coli* under HCDC (Heggeset *et al*., 2013; Kucharova *et al*., 2013; Tøndervik *et al*., 2013).

## Application of XylS/*Pm* for fine‐tuned regulated expression of genes and gene clusters in many different bacterial species

XylS/*Pm* displays many favourable properties, which make it a valuable tool for metabolic engineering and other purposes which require fine‐tuning of expression of genes or gene clusters, usually at physiologically relevant levels. The expression system has been shown to function well in a wide range of different Gram‐negative organisms, and recently also in Gram‐positive species. The possibility to use XylS/*Pm* for fine‐tuning of expression is a consequence of the nearly proportional relation between the expression level and the concentration of the inducer. In some cases, even the uninduced expression level from *Pm* may be higher than desired, emphasizing the need for mutants that exhibit reduced background expression while still being inducible. A complete list of all reported bacterial species in which the XylS/*Pm* system has been applied is presented in Table 2 and selected examples are outlined below. Due to its applicability to a wide range of bacterial hosts, XylS/*Pm* is available in the SEVA‐DB as one of the broad‐host‐range expression cargos formatted following the SEVA standard to allow combination of the system with other optimal plasmid elements (SEVA‐DB, http://seva.cnb.csic.es; Silva‐Rocha *et al*., 2013).

**Table 2:** Host organisms used for the XylS/*Pm*‐mediated expression of heterologous proteins and examples of applications in these hosts

Host organism	Applications and characteristics	Selected references
*Acinetobacter calcoaceticus*	Assay of catechol 2,3‐dioxygenase activities	Mermod *et al*. (1986)
*Aerobacter aerogenes*	Assay of catechol 2,3‐dioxygenase activities	Mermod *et al*. (1986)
*Aeromonas hydrophila*	Assay of β‐galactosidase and catechol 2,3‐dioxygenase activities	Ramos *et al*. (1988); Mermod *et al*. (1986)
*Agrobacterium tumefaciens*	Targetrons expression, assay of catechol 2,3‐dioxygenase activities; high‐level expression	Mermod *et al*. (1986), Yao and Lambowitz (2007)
*Alcaligenes eutrophus*	Assay of catechol 2,3‐dioxygenase activities	Mermod *et al*. (1986)
*Azotobacter vinelandii*	Over‐expression of NifH (Fe protein), AlgE3 expression, assay of β‐galactosidase activities	Steigedal *et al*. (2008), Ramos *et al*. (1988), Nag and Pal (2013)
*Brucella abortus*	Mutant strains generation and evaluation; high‐level, tightly controlled expression, versatility	Ortiz‐Román *et al*. (2014)
*Comamonas* sp.	*ipha* expression, *pmdB* expression, terephthalate degradation gene cluster expression	Sasoh *et al*. (2006), Fukuhara *et al*. (2010), Kamimura *et al*. (2010)
*Erwinia carotovora*	Assay of catechol 2,3‐dioxygenase activities	Mermod *et al*. (1986)
*Escherichia coli*	*pfa* biosynthetic gene cluster expression, E1 and E2 glycoproteins production, carotenoid production, targetrons expression; high‐level recombinant production, fine‐tuning	Yao and Lambowitz (2007), Zhuang *et al*. (2009), Netzer *et al*. (2010), Tøndervik *et al*. (2013), Gemperlein *et al*. (2016)
*Hyphomicrobium* sp.	Assay of catechol 2,3‐dioxygenase activities	Mermod *et al*. (1986)
*Klebsiella pneumoniae*	Assay of catechol 2,3‐dioxygenase activities	Mermod *et al*. (1986)
*Moraxella nonliquefaciens*	Assay of catechol 2,3‐dioxygenase activities	Mermod *et al*. (1986)
*Mycobacterium smegmatis*	Adaptation of the XylS/*Pm* for expression in Mycobacteria; expression of firefly luciferase genes tight control, high‐level expression	Dragset *et al*. (2015)
*Mycobacterium tuberculosis*	Adaptation of the XylS/*Pm* for expression in Mycobacteria; expression of firefly luciferase genes tight control, high‐level expression	Dragset *et al*. (2015)
*Myxococcus xanthus*	Myxothiazol gene cluster expression	Perlova *et al*. (2006)
*Paracoccus denitrificans*	Assay of catechol 2,3‐dioxygenase activities	Mermod *et al*. (1986)
*Pseudomonas aeruginosa*	Expression of genes involved in biofilm‐specific antibiotic resistance, expression of genes involved in ethanol oxidation, targetrons expression, 3‐oxosteroid D1‐dehydrogenase gene expression, over‐expression of *oprH*; high‐level expression	Bell *et al*. (1991), Plesiat *et al*. (1997), Yao and Lambowitz (2007), Zhang and Mah (2008), (Zhang *et al*., 2011, 2013); Beaudoin *et al*. (2012)
*Pseudomonas entomophila*	*ndhSL* gene cluster expression; tight control	Yang *et al*. (2009)
*Pseudomonas fluorescens*	*gfpmut3* expression, assay of alginate synthesis, *adnA* expression; fine‐tuning	Casaz *et al*. (2001), Boldt *et al*. (2004), Bakkevig *et al*. (2005), Liu *et al*. (2010)
*Pseudomonas oleovorans*	Polyhydroxyalkanoate production; stable regulation	Prieto *et al*. (1999)
*Pseudomonas putida*	Minitransposon delivery vector construction, recombinant antibody fragments production, I‐SceI genomic deletions system, quinoline 2‐oxidoreductase expression, metallothioneins production; expression of firefly luciferase genes tight control, low cost	Purvanov and Fetzner (2005), Dammeyer *et al*. (2011), Martínez‐García and de Lorenzo (2011), Nikel and de Lorenzo (2013)
*Pseudomonas stutzeri*	Different fusion proteins expression, expression of lysis gene E	Kloos *et al*. (1994), Gross *et al*. (2005, 2006)
*Pseudomonas syringae*	Different fusion proteins expression	Gross *et al*. (2005, 2006)
*Pseudomonas taiwanensis*	I‐SceI genomic deletions system; tight control	Volmer *et al*. (2014), Schmutzler *et al*. (2015)
*Pseudomonas testosteroni*	Assay of catechol 2,3‐dioxygenase activities	Mermod *et al*. (1986)
*Ralstonia eutropha*	Development of genetic toolbox for *Ralstonia eutropha*, mouse metallothionein I protein production; tight control, high‐level expression	Valls *et al*. (2000), Bi *et al*. (2013)
*Salmonella enterica*	Cytosine deaminase expression, virulescence factor expression, *nasF* and dTomato RFP expression, *codA*, lacZ, *trp* and *gfp* expression; high‐level expression	Royo *et al*. (2007), Medina *et al*. (2011), Mesa‐Pereira *et al*. (2013, 2015)
*Serratia marcescens*	Assay of catechol 2,3‐dioxygenase activities	Mermod *et al*. (1986)
*Shigella flexneri*	Assay of β‐galactosidase activities	Ramos *et al*. (1988)
*Spingomonas paucimobilis*	Suicide system control; tight regulation	Lan *et al*. (2014)
*Vibrio cholerae*	Expression of lysis gene E	Eko *et al*. (2000, 2003, 2014), Ramey *et al*. (2009)
*Xanthomonas campestris*	Phosphoglucomutase expression, assay of catechol 2,3‐dioxygenase activities; high‐level expression, tight regulation	Mermod *et al*. (1986), Blatny *et al*. (1997a,b)

### Broad‐host‐range applications of the wild‐type XylS*/Pm* expression cassette

The *xanA* gene of *Xanthomonas campestris* encodes a bifunctional phosphogluco‐mannomutase required for biosynthesis of the commercially important polysaccharide xanthan (Köplin *et al*., 1992). By expressing *xanA* from XylS/*Pm* in a *xanA*‐deficient *X. campestris* host, the synthesis of xanthan could be monitored in induced and uninduced cells. There was virtually no xanthan synthesis in the absence of inducer, while polymer synthesis was activated to wild‐type levels upon induction of the *Pm* promoter. The XanA production under these conditions was not limiting for xanthan biosynthesis in *X. campestris* (Winther‐Larsen *et al*., 2000a).

Martínez‐García and de Lorenzo (2011) employed a procedure involving two basic plasmid architectures aiming for multiple markerless gene replacements in a range of different of Gram‐negative bacterial species including *P. putida*. One plasmid was responsible for introducing I‐SceI site(s) within the target genome region through homologous recombination between plasmid‐encoded DNA and the chromosome. The second plasmid provided conditional expression of the I‐SceI endonuclease upon activation of XylS/*Pm* in a manner that does not depend on the cellular growth phase (Martínez‐García and de Lorenzo, 2011). The results obtained proved the effectiveness of the I‐SceI methodology which later allowed for introduction of targeted deletions into 11 chromosomal regions (comprising 300 genes) of *P. putida* and significantly improved the growth properties of the resulting recombinant strain (Martínez‐García *et al*., 2014). Chromosome cleavage with unique I‐SceI sites and XylS/*Pm*‐controlled expression of the target enzyme were also applied to eliminate operons encoding anthranilate phosphoribosyltransferase, indole‐3‐glycerol phosphate synthase and chorismate mutase and to establish anthranilate production in *P. putida* (Kuepper *et al*., 2015).

The XylS/*Pm*‐based reporter system was used to control expression of a fluorescent protein denoted EcFbFp (*E. coli* – optimized flavin mononucleotide‐based fluorescent protein) and alkyl halide degradation operon from *Pseudomonas pavonaceae* responsible for organohalide metabolism. Successful expression and resulting activity of these proteins confirmed the capabilities of the recombinant strain to grow under anoxic conditions (Nikel and de Lorenzo, 2013).

Martínez *et al*. (2011) reported the construction of a programmed self‐disruptive *P. putida* BXHL strain that facilitates the release of polyhydroxyalkanoic acid (PHA) granules to the extracellular medium. This is biotechnologically important as an efficient PHA recovery process is essential to reduce the cost of microbial PHA production. The engineered system was based on two proteins from the pneumococcal bacteriophage EJ‐1, Ejh holin and Ejl endolysin, and the corresponding *ejh* and *ejl* genes were inserted into the chromosome of a *tolB* mutant of *P. putida* KT2440 under control of the XylS/*Pm*. The *tolB* gene encodes a periplasmic protein and a mutation in this gene causes alternations in the outer membrane stability. With this expression system, cell lysis could be controlled by using 3‐methylbenzoate as inducer.

Valls *et al*. (2000) described the construction of a *R. eutropha* strain with an enhanced ability to immobilize Cd^2+^ ions from the external media. The effect was observed as a result of stable chromosomal integration of the minitransposon TnMTβ‐1 containing the *mtb* gene placed downstream of the *Pm* promoter. This cassette allowed for expression of the mouse metallothionein I (MT) protein fused to the autotransporter β‐domain (MTβ) of the IgA protease of *Neisseria gonorrhoeae*. Production of MTβ was found to be strictly dependent on the presence of 3‐methylbenzoate in the growth medium, thus demonstrating the tight control of the *Pm* promoter in *R. eutropha*.

In *Myxococcus xanthus,* the myxothiazol biosynthetic gene cluster *mta* originating from *Stigmatella aurantiaca* was placed under control of the XylS/*Pm* system and integrated into the *M. xanthus* chromosome (Perlova *et al*., 2006). The resulting recombinant strain produced myxothiazol in yields comparable to the natural *S. aurantiaca* producer strain. XylS/*Pm* was also used for controlled expression of genes involved in biosynthesis of secondary metabolites that may be toxic for the host strain. Heterologous expression of the myxochromide S cluster from *S. aurantiaca* in a *P. putida* mutant strain resulted in high myxochromide production levels in the recombinant cells (Wenzel *et al*., 2005).

Interestingly, the XylS/*Pm* expression system was recently also demonstrated to function in Gram‐positive bacteria (Dragset *et al*., 2015). By making some necessary modifications to the XylS/*Pm* regulated gene expression vector, robust time‐ and dose‐dependent reversible induction accompanied by low background expression levels was obtained in both *Mycobacterium smegmatis* and *Mycobacterium tuberculosis* (Table 2). This result should open up opportunities for exploring the application of the XylS/*Pm* expression system also in other Gram‐positive bacteria.

In the RK2‐based broad‐host‐range expression vectors harbouring XylS*/Pm* (see above), plasmid replication relies on the initiation protein TrfA encoded by the *trfA* gene located on the vectors (Blatny *et al*., 1997a). A plasmid denoted pJBSD1 was constructed with a mutant version of the *trfA* gene placed under control of *Pm*, and this plasmid was demonstrated to be dependent on a *Pm* inducer to replicate in *E. coli*. This plasmid can be used as a conditional suicide vector system for targeted chromosomal integration via homologous recombination in *E*. coli and potentially also in other Gram‐negative bacteria (Karunakaran *et al*., 1999).

XylS2 is a mutant derivative of XylS that can be activated by salicylic acid (Ramos *et al*., 1986). The *xylS2* gene together with *Pm* was used as key components in the construction of a novel regulatory circuit demonstrated to be useful in *Salmonella* bacteria with two modules operating in cascade (Royo *et al*., 2007). More specifically, the expression of *xylS2* was coupled to the NahR‐dependent *Psal* promoter, and the cassette was inserted into the bacterial chromosome. NahR is a transcriptional activator that can be induced by salicylate and then promotes transcription from *Psal*. This genetic background was then used in a host for the expression of plasmid‐borne reporter genes placed under the control of the *Pm* promoter. In the absence of salicylate, XylS expression levels were low and XylS was not active, and accordingly expression of reporter genes from *Pm* was very low. In the presence of salicylic acid, NahR activated transcription from *Psal* and thus produced XylS2, which then subsequently bound the effector molecule salicylic acid, becoming activated and causing synergistically increased transcription from *Pm*. The system was demonstrated to be useful for studying bacterium–host interactions *in vivo* in both mouse and tumour cells by expressing the GFP protein from the *Pm* promoter (for a review, see Becker *et al*., 2010). Later the circuit has been modified and improved by using different replicons for the *Pm* expression module (Medina *et al*., 2011). This has been useful for *in vivo* studies of *Salmonella* upon infection of different eukaryotic cells (Mesa‐Pereira *et al*., 2013).

### Application of XylS/*Pm* mutant derivatives

Totally 12 different derivatives of the XylS/*Pm* expression cassette with alterations in the *rbs* coding region (denoted SD mutants; see above) were used for fine‐tuned low‐level expression of a heterologous phosphoglucomutase (Pgm) in a *pgm*‐deficient mutant of *E. coli* growing in the presence of galactose (Brautaset *et al*., 1998, 2000). Galactose enters the cells eventually as G‐1‐P and can be channelled into catabolism by the action of Pgm. In the absence of Pgm, G‐1‐P accumulation leads to biosynthesis of intracellular amylose. The recombinant cells were cultivated without any induction, and Pgm activity was downregulated up to 51‐fold when using the SD mutants compared with the wild‐type XylS/*Pm*. In this way, amylose accumulation in the respective cells could be gradually varied demonstrating that very low expression levels may be needed to obtain full control of metabolic pathway activities. The induction ratios of mutant derivatives were also shown to be strongly affected compared with the wild‐type XylS/*Pm* cassette (Winther‐Larsen *et al*., 2000b).

XylS/*Pm* was also employed to modulate production, composition and localization of biosynthesis and export components of the important biopolymer alginate in *P. fluorescens*. This was achieved by controlled expression of the mannuronan C‐5‐epimerase gene *algG* (Gimmestad *et al*., 2003), the alginate lyase gene *algL* (Bakkevig *et al*., 2005) and the porin gene *algE* (Maleki *et al*., 2016) respectively. In the latter example, the density of alginate secretion components in the cell membrane could be modulated by using the unique properties of XylS/Pm for regulated low expression. In all these cases, a combination of chromosomal integration approach and a specific *Pm* promoter mutant denoted *PmG5* was used to ensure physiological relevant low expression of the respective genes. *PmG5* provides lower background expression in the absence of inducer than *Pm* wild type in *P. fluorescens* (Gimmestad *et al*., 2003).

XylS/*Pm* and its mutant derivatives were effectively used for controlled and functional expression of the biosynthetic gene cluster of the C50 carotenoid sarcinaxanthin originating from *Micrococcus luteus*, enabling efficient sarcinaxanthin production in *E. coli* (Netzer *et al*., 2010). The gene cluster includes totally seven protein coding sequences and substitution of single genes with heterologous genes allowed for production of unnatural C50 carotenoids in *E. coli*. By using certain *Pm* 5′‐UTR down mutants, it was later demonstrated that the XylS*/Pm* system could be used to control the sarcinaxanthin production level in recombinant *E. coli* cells and in this way metabolic bottlenecks in the sarcinaxanthin biosynthetic pathway could be identified (Lale *et al*., 2011). Recently, one of these 5′‐UTR region modifications allowed to reduce the leakiness of the XylS/*Pm* system which was used in combination with *PT7lac* promoter to investigate potential of two *Pseudomonas* spp. strains for low‐temperature expression of a red fluorescent reporter protein (mCherry) (Bjerga *et al*., 2016).

Gemperlein *et al*. (2016) employed a specific 5′‐UTR mutated version of the *Pm* promoter region for expression of the *pfa* biosynthetic gene cluster originating from *Aetherobacter fasciculatus* in a *P. putida* KT2440 host strain. The *pfa* gene cluster encodes a set of myxobacterial polyunsaturated fatty acid (PUFA) synthases which are polyketide synthase‐like enzymes catalysing biosynthesis of long‐chain (LC) PUFAs in *A. fasciculatus*. The recombinant strain was further engineered for co‐expression of the *afppt* gene from *A. fasciculatus* encoding a phosphopantetheinyl transferase, proposed to catalyse phosphopantetheinylation of the PUFA synthases. The *afppt* gene was placed under the control of a separate XylS/*Pm* cassette and integrated into the host chromosome. Induced recombinant expression in recombinant *P. putida* KT2440 strain resulted in 3‐fold increased LC‐PUFA production yield compared with the wild‐type strain.

## Conclusions

The inducible *Pm* promoter together with its cognate positive regulator XylS display many favourable properties that makes the XylS/*Pm* system highly valuable for different applications related to recombinant gene expression. The cassette can be used in a wide range of different Gram‐negative bacteria and it was recently also demonstrated to function in Gram‐positive organisms further extending the range of its potential applications. The system is characterized by the simple mode of regulation which can be achieved by using different inducers which are typically non‐metabolized by a host and enter passively to the cells. These properties together with a dose‐dependent induction response and low background expression in the absence of inducer make this expression system highly flexible for both high‐level protein production and metabolic engineering in many host organisms. Synthetic biology continuously raises increasing need for useful expression systems, enabling fine‐tuned expression of genes and gene cluster around physiologically relevant levels. Ongoing bioprospecting and advanced genetic engineering aim to generate synthetic gene clusters for microbial production of complex chemicals, such as antibiotics, biopolymers and terpenoids, for various medical and industrial applications. To fully explore the potential of such approaches, genetic tools enabling the functional expression of the desired genes in the preferred microbial host will be crucial. The combinatorial mutagenesis efforts made to improve and expand the properties of XylS/*Pm* have provided better tools for such purposes, and the technologies used to improve this particular system, as presented here, have great potentials to be used for alternative bacterial expression systems.

## Conflict of interest

None declared.

## References

[mbt212701-bib-0001] Abril, M.A. , Michan, C. , Timmis, K.N. , and Ramos, J.L. (1989) Regulator and enzyme specificities of the TOL plasmid‐encoded upper pathway for degradation of aromatic hydrocarbons and expansion of the substrate range of the pathway. J Bacteriol 171: 6782–6790. 2687253 10.1128/jb.171.12.6782-6790.1989 PMC210577

[mbt212701-bib-0002] Aune, T.E. , Bakke, I. , Drabløs, F. , Lale, R. , Brautaset, T. , and Valla, S. (2010) Directed evolution of the transcription factor XylS for development of improved expression systems. Microb Biotechnol 3: 38–47. 21255304 10.1111/j.1751-7915.2009.00126.x PMC3815945

[mbt212701-bib-0003] Bakke, I. , Berg, L. , Aune, T.E. , Brautaset, T. , Sletta, H. , Tøndervik, A. , and Valla, S. (2009) Random mutagenesis of the Pm promoter as a powerful strategy for improvement of recombinant‐gene expression. Appl Environ Microbiol 75: 2002–2011. 19201973 10.1128/AEM.02315-08 PMC2663224

[mbt212701-bib-0004] Bakkevig, K. , Sletta, H. , Gimmestad, M. , Aune, R. , Ertesvåg, H. , Degnes, K. , *et al* (2005) Role of the *Pseudomonas fluorescens* alginate lyase (AlgL) in clearing the periplasm of alginates not exported to the extracellular environment. J Bacteriol 187, 8375–8384. 16321942 10.1128/JB.187.24.8375-8384.2005 PMC1317005

[mbt212701-bib-0005] Balzer, S. , Kucharova, V. , Megerle, J. , Lale, R. , Brautaset, T. , and Valla, S. (2013) A comparative analysis of the properties of regulated promoter systems commonly used for recombinant gene expression in *Escherichia coli* . Microb Cell Fact 12: 26. 23506076 10.1186/1475-2859-12-26 PMC3621392

[mbt212701-bib-0006] Beaudoin, T. , Zhang, L. , Hinz, A.J. , Parr, C.J. , and Mah, T.‐F. (2012) The biofilm‐specific antibiotic resistance gene *ndvB* is important for expression of ethanol oxidation genes in *Pseudomonas aeruginosa* biofilms. J Bacteriol 194: 3128–3136. 22505683 10.1128/JB.06178-11 PMC3370848

[mbt212701-bib-0216] Becker, P.D. , Royo, J.L. , and Guzman, C.A. (2010) Exploitation of prokaryotic expression systems based on the salicylate‐dependent control circuit encompassing nahR/Psal::xylS2 for biotechnological applications. *Bioeng Bugs* 1: 244–251. 10.4161/bbug.1.4.11247 PMC3026463 21327056

[mbt212701-bib-0007] Bell, A. , Bains, M. , and Hancock, R.E. (1991) *Pseudomonas aeruginosa* outer membrane protein OprH: expression from the cloned gene and function in EDTA and gentamicin resistance. J Bacteriol 173: 6657–6664. 1938872 10.1128/jb.173.21.6657-6664.1991 PMC209012

[mbt212701-bib-0008] Berg, L. , Lale, R. , Bakke, I. , Burroughs, N. , and Valla, S. (2009) The expression of recombinant genes in *Escherichia coli* can be strongly stimulated at the transcript production level by mutating the DNA‐region corresponding to the 5′‐untranslated part of mRNA. Microb Biotechnol 2: 379–389. 21261932 10.1111/j.1751-7915.2009.00107.x PMC3815758

[mbt212701-bib-0009] Berg, L. , Kucharova, V. , Bakke, I. , Valla, S. , and Brautaset, T. (2012) Exploring the 5′‐UTR DNA region as a target for optimizing recombinant gene expression from the strong and inducible Pm promoter in *Escherichia coli* . J Biotechnol 158: 224–230. 21801767 10.1016/j.jbiotec.2011.07.012

[mbt212701-bib-0010] Bi, C. , Su, P. , Müller, J. , Yeh, Y.D. , Chhabra, S.R. , Beller, H.R. , *et al* (2013) Development of a broad‐host synthetic biology toolbox for *Ralstonia eutropha* and its application to engineering hydrocarbon biofuel production. Microb Cell Fact 12: 107. 24219429 10.1186/1475-2859-12-107 PMC3831590

[mbt212701-bib-0011] Binder, D. , Probst, C. , Grünberger, A. , Hilgers, F. , Loeschcke, A. , Jaeger, K.‐E. , *et al* (2016) Comparative single‐cell analysis of different *E. coli* expression systems during microfluidic cultivation. PLoS ONE 11: e0160711. 27525986 10.1371/journal.pone.0160711 PMC4985164

[mbt212701-bib-0012] Bjerga, G.E.K. , Lale, R. , and Williamson, A.K. (2016) Engineering low‐temperature expression systems for heterologous production of cold‐adapted enzymes. Bioengineered 7: 33–38. 26710170 10.1080/21655979.2015.1128589 PMC4878266

[mbt212701-bib-0013] Blatny, J.M. , Brautaset, T. , Winther‐Larsen, H.C. , Haugan, K. , and Valla, S. (1997a) Construction of a versatile set of broad‐host‐range cloning and expression vectors based on the RK2 replicon. Appl Environ Microbiol 63: 370–379. 9023917 10.1128/aem.63.2.370-379.1997 PMC168329

[mbt212701-bib-0014] Blatny, J.M. , Brautaset, T. , Winther‐Larsen, H.C. , Karunakaran, P. , and Valla, S. (1997b) Improved broad‐host‐range RK2 vectors useful for high and low regulated gene expression levels in gram‐negative bacteria. Plasmid 38: 35–51. 9281494 10.1006/plas.1997.1294

[mbt212701-bib-0015] Boldt, T.S. , Sørensen, J. , Karlson, U. , Molin, S. , and Ramos, C. (2004) Combined use of different Gfp reporters for monitoring single‐cell activities of a genetically modified PCB degrader in the rhizosphere of alfalfa. FEMS Microbiol Ecol 48: 139–148. 19712397 10.1016/j.femsec.2004.01.002

[mbt212701-bib-0016] Brautaset, T. , Standal, R. , Espevik, F. , and Valla, S. (1994) Nucleotide sequence and expression analysis of the *Acetobacter xylinum* phosphoglucomutase gene. Microbiology 140: 1183–1188. 8025683 10.1099/13500872-140-5-1183

[mbt212701-bib-0017] Brautaset, T. , Petersen, S. , and Valla, S. (1998) An experimental study on carbon flow in *Escherichia coli* as a function of kinetic properties and expression levels of the enzyme phosphoglucomutase. Biotechnol Bioeng 58: 299–302. 10191405

[mbt212701-bib-0018] Brautaset, T. , Petersen, S.B. , and Valla, S. (2000) *In vitro* determined kinetic properties of mutant phosphoglucomutases and their effects on sugar metabolism in *Escherichia coli* . Metab Eng 2: 104–114. 10935726 10.1006/mben.1999.0145

[mbt212701-bib-0019] Brautaset, T. , Lale, R. , and Valla, S. (2009) Positively regulated bacterial expression systems. Microb Biotechnol 2: 15–30. 21261879 10.1111/j.1751-7915.2008.00048.x PMC3815419

[mbt212701-bib-0020] Casaz, P. , Happel, A. , Keithan, J. , Read, D.L. , Strain, S.R. , and Levy, S.B. (2001) The *Pseudomonas fluorescens* transcription activator AdnA is required for adhesion and motility. Microbiology 147: 355–361. 11158352 10.1099/00221287-147-2-355

[mbt212701-bib-0021] Cebolla, A. , Sousa, C. , and de Lorenzo, V. (2001) Rational design of a bacterial transcriptional cascade for amplifying gene expression capacity. Nucleic Acids Res 29: 759–766. 11160899 10.1093/nar/29.3.759 PMC30378

[mbt212701-bib-0022] Cebolla, A. , Royo, J.L. , de Lorenzo, V. , and Santero, E. (2002) Improvement of recombinant protein yield by a combination of transcriptional amplification and stabilization of gene expression. Appl Environ Microbiol 68: 5034–5041. 12324354 10.1128/AEM.68.10.5034-5041.2002 PMC126411

[mbt212701-bib-0023] Dammeyer, T. , Steinwand, M. , Krüger, S.‐C. , Dübel, S. , Hust, M. , and Timmis, K.N. (2011) Efficient production of soluble recombinant single chain Fv fragments by a *Pseudomonas putida *strain KT2440 cell factory. Microb Cell Fact 10: 11. 21338491 10.1186/1475-2859-10-11 PMC3053225

[mbt212701-bib-0060] Domínguez‐Cuevas, P. , Marín, P. , Ramos, J.M. , and Marqués, S. (2005) RNA polymerase holoenzymes can share a single transcription start site for the *Pm* promoter. Critical nucleotides in the ‐7 to ‐18 region are needed to select between RNA polymerase with sigma38 or sigma32. J Biol Chem 280: 41315–41323. 16230361 10.1074/jbc.M505415200

[mbt212701-bib-0024] Domínguez‐Cuevas, P. , Marín, P. , Marqués, S. , and Ramos, J.L. (2008) XylS–Pm promoter interactions through two helix–turn–helix motifs: identifying XylS residues important for DNA binding and activation. J Mol Biol 375: 59–69. 18005985 10.1016/j.jmb.2007.10.047

[mbt212701-bib-0025] Domínguez‐Cuevas, P. , Ramos, J.L. , and Marqués, S. (2010) Sequential XylS‐CTD binding to the Pm promoter induces DNA bending prior to activation. J Bacteriol 192: 2682–2690. 20363935 10.1128/JB.00165-10 PMC2876475

[mbt212701-bib-0026] Dragset, M.S. , Barczak, A.K. , Kannan, N. , Mærk, M. , Flo, T.H. , Valla, S. , *et al* (2015) Benzoic acid‐inducible gene expression in mycobacteria. PLoS ONE 10: e0134544. 26348349 10.1371/journal.pone.0134544 PMC4562662

[mbt212701-bib-0027] Egan, S.M. (2002) Growing repertoire of AraC/XylS activators. J Bacteriol 184: 5529–5532. 12270809 10.1128/JB.184.20.5529-5532.2002 PMC139625

[mbt212701-bib-0028] Eko, F.O. , Mayr, U.B. , Attridge, S.R. , and Lubitz, W. (2000) Characterization and immunogenicity of *Vibrio cholerae* ghosts expressing toxin‐coregulated pili. J Biotechnol 83: 115–123. 11000467 10.1016/s0168-1656(00)00315-1

[mbt212701-bib-0029] Eko, F.O. , Lubitz, W. , McMillan, L. , Ramey, K. , Moore, T.T. , Ananaba, G.A. , *et al* (2003) Recombinant *Vibrio cholerae* ghosts as a delivery vehicle for vaccinating against *Chlamydia trachomatis* . Vaccine 21: 1694–1703. 12639492 10.1016/s0264-410x(02)00677-1

[mbt212701-bib-0030] Eko, F.O. , Mania‐Pramanik, J. , Pais, R. , Pan, Q. , Okenu, D.M.N. , Johnson, A. , *et al* (2014) *Vibrio cholerae* ghosts (VCG) exert immunomodulatory effect on dendritic cells for enhanced antigen presentation and induction of protective immunity. BMC Immunol 15, 584. 25551828 10.1186/s12865-014-0056-x PMC4312469

[mbt212701-bib-0031] Frerichs‐Deeken, U. , Goldenstedt, B. , Gahl‐Janssen, R. , Kappl, R. , Hüttermann, J. , and Fetzner, S. (2003) Functional expression of the quinoline 2‐oxidoreductase genes (*qorMSL*) in *Pseudomonas putida* KT2440 pUF1 and in *P. putida* 86‐1 Δ*qor* pUF1 and analysis of the Qor proteins. Eur J Biochem 270: 1567–1577. 12654012 10.1046/j.1432-1033.2003.03526.x

[mbt212701-bib-0032] Fukuhara, Y. , Inakazu, K. , Kodama, N. , Kamimura, N. , Kasai, D. , Katayama, Y. , *et al* (2010) Characterization of the isophthalate degradation genes of *Comamonas* sp. strain E6. Appl Environ Microbiol 76: 519–527. 19933340 10.1128/AEM.01270-09 PMC2805221

[mbt212701-bib-0033] Gallegos, M.T. , Marqués, S. , and Ramos, J.L. (1996) Expression of the TOL plasmid *xylS* gene in *Pseudomonas putida* occurs from an alpha 70‐dependent promoter or from alpha 70‐ and alpha 54‐dependent tandem promoters according to the compound used for growth. J Bacteriol 178: 2356–2361. 8636038 10.1128/jb.178.8.2356-2361.1996 PMC177945

[mbt212701-bib-0034] Gallegos, M.‐T. , Schleif, R. , Bairoch, A. , Hofmann, K. , and Ramos, J.L. (1997) AraC/XylS family of transcriptional regulators. Microbiol Mol Biol Rev 61: 393–410. 9409145 10.1128/mmbr.61.4.393-410.1997 PMC232617

[mbt212701-bib-0035] Gemperlein, K. , Zipf, G. , Bernauer, H.S. , Müller, R. , and Wenzel, S.C. (2016) Metabolic engineering of *Pseudomonas putida* for production of docosahexaenoic acid based on a myxobacterial PUFA synthase. Metab Eng 33: 98–108. 26617065 10.1016/j.ymben.2015.11.001

[mbt212701-bib-0036] Gimmestad, M. , Sletta, H. , Ertesvaag, H. , Bakkevig, K. , Jain, S. , Skjåk‐Bræk, G. , *et al* (2003) The *Pseudomonas fluorescens* AlgG protein, but not its mannuronan C‐5‐epimerase activity, is needed for alginate polymer function. J Bacteriol 185: 3515–3523. 12775688 10.1128/JB.185.12.3515-3523.2003 PMC156231

[mbt212701-bib-0037] Golomb, M. , and Chamberlin, M. (1974) Characterization of T7‐specific ribonucleic acid polymerase. J Biol Chem 249: 2858–2868. 4828324

[mbt212701-bib-0038] González‐Pérez, M.M. , Ramos, J.L. , Gallegos, M.T. , and Marqués, S. (1999) Critical nucleotides in the upstream region of the XylS‐dependent TOL *meta*‐cleavage pathway operon promoter as deduced from analysis of mutants. J Biol Chem 274: 2286–2290. 9890992 10.1074/jbc.274.4.2286

[mbt212701-bib-0039] González‐Pérez, M.M. , Marqués, S. , Domínguez‐Cuevas, P. , and Ramos, J.L. (2002) XylS activator and RNA polymerase binding sites at the *Pm* promoter overlap. FEBS Lett 519: 117–122. 12023029 10.1016/s0014-5793(02)02730-8

[mbt212701-bib-0040] Greated, A. , Lambertsen, L. , Williams, P.A. , and Thomas, C.M. (2002) Complete sequence of the IncP‐9 TOL plasmid pWW0 from *Pseudomonas putida* . Environ Microbiol 4: 856–871. 12534468 10.1046/j.1462-2920.2002.00305.x

[mbt212701-bib-0041] Gross, F. , Gottschalk, D. , and Müller, R. (2005) Posttranslational modification of myxobacterial carrier protein domains in *Pseudomonas* sp. by an intrinsic phosphopantetheinyl transferase. Appl Microbiol Biotechnol 68: 66–74. 15635461 10.1007/s00253-004-1836-7

[mbt212701-bib-0042] Gross, F. , Luniak, N. , Perlova, O. , Gaitatzis, N. , Jenke‐Kodama, H. , Gerth, K. , *et al* (2006) Bacterial type III polyketide synthases: phylogenetic analysis and potential for the production of novel secondary metabolites by heterologous expression in pseudomonads. Arch Microbiol 185: 28–38. 16395556 10.1007/s00203-005-0059-3

[mbt212701-bib-0043] Heggeset, T.M. , Kucharova, V. , Nærdal, I. , Valla, S. , Sletta, H. , Ellingsen, T.E. , and Brautaset, T. (2013) Combinatorial mutagenesis and selection of improved signal sequences and their application for high‐level production of translocated heterologous proteins in *Escherichia coli* . Appl Environ Microbiol 79: 559–568. 23144128 10.1128/AEM.02407-12 PMC3553763

[mbt212701-bib-0044] Inouye, S. , Nakazawa, A. , and Nakazawa, T. (1987) Expression of the regulatory gene *xylS* on the TOL plasmid is positively controlled by the *XylR* gene product. Proc Natl Acad Sci USA 84: 5182–5186. 2440045 10.1073/pnas.84.15.5182 PMC298818

[mbt212701-bib-0045] Jiménez, J.I. , Pérez‐Pantoja, D. , Chavarría, M. , Díaz, E. , and de Lorenzo, V. (2014) A second chromosomal copy of the *catA* gene endows *Pseudomonas putida* mt‐2 with an enzymatic safety valve for excess of catechol. Environ Microbiol 16: 1767–1778. 24341396 10.1111/1462-2920.12361

[mbt212701-bib-0046] Kaldalu, N. , Toots, U. , de Lorenzo, V. , and Ustav, M. (2000) Functional domains of the TOL plasmid transcription factor XylS. J Bacteriol 182: 1118–1126. 10648539 10.1128/jb.182.4.1118-1126.2000 PMC94389

[mbt212701-bib-0047] Kamimura, N. , Aoyama, T. , Yoshida, R. , Takahashi, K. , Kasai, D. , Abe, T. , *et al* (2010) Characterization of the protocatechuate 4,5‐cleavage pathway operon in *Comamonas* sp. strain E6 and discovery of a novel pathway gene. Appl Environ Microbiol 76: 8093–8101. 20952641 10.1128/AEM.01863-10 PMC3008244

[mbt212701-bib-0048] Karunakaran, P. , Endresen, D.T. , Ertesvaag, H. , Janet Martha Blatny, J.M. and Valla, S. (1999) A small derivative of the broad‐host‐range plasmid RK2 which can be switched from a replicating to a non‐replicating state as a response to an externally added inducer. FEMS Microbiol Lett 180, 221–227. 10556715 10.1111/j.1574-6968.1999.tb08799.x

[mbt212701-bib-0049] Kessler, B. , de Lorenzo, V. , and Timmis, K.N. (1993) Identification of a *cis*‐acting sequence within the *Pm* promoter of the TOL plasmid which confers XylS‐mediated responsiveness to substituted benzoates. J Mol Biol 230: 699–703. 8478926 10.1006/jmbi.1993.1189

[mbt212701-bib-0050] Kessler, B. , Timmis, K. , and de Lorenzo, V. (1994) The organization of the *Pm* promoter of the TOL plasmid reflects the structure of its cognate activator protein XylS. Molec Gen Genet 244: 596–605. 7969028 10.1007/BF00282749

[mbt212701-bib-0051] Kloos, D.U. , Strätz, M. , Güttler, A. , Steffan, R.J. , and Timmis, K.N. (1994) Inducible cell lysis system for the study of natural transformation and environmental fate of DNA released by cell death. J Bacteriol 176: 7352–7361. 7961508 10.1128/jb.176.23.7352-7361.1994 PMC197125

[mbt212701-bib-0052] Köplin, R. , Arnold, W. , Hotte, B. , Simon, R. , Wang, G. , and Puhler, A. (1992) Genetics of xanthan production in *Xanthomonas campestris*: the *xanA* and *xanB* genes are involved in UDP‐glucose and GDP‐mannose biosynthesis. J Bacteriol 174: 191–199. 1370280 10.1128/jb.174.1.191-199.1992 PMC205695

[mbt212701-bib-0053] Kucharova, V. , Skancke, J. , Brautaset, T. , and Valla, S. (2013) Design and optimization of short DNA sequences that can be used as 5′ fusion partners for high‐level expression of heterologous genes in *Escherichia coli* . Appl Environ Microbiol 79: 6655–6664. 23974137 10.1128/AEM.01676-13 PMC3811499

[mbt212701-bib-0054] Kuepper, J. , Dickler, J. , Biggel, M. , Behnken, S. , Jäger, G. , Wierckx, N. , and Blank, L.M. (2015) Metabolic engineering of *Pseudomona putida* KT2440 to produce anthranilate from glucose. Front Microbiol 6: 1310. 26635771 10.3389/fmicb.2015.01310 PMC4656820

[mbt212701-bib-0055] Lale, R. , Berg, L. , Stüttgen, F. , Netzer, R. , Stafsnes, M. , Brautaset, T. , *et al* (2011) Continuous control of the flow in biochemical pathways through 5′ untranslated region sequence modifications in mRNA expressed from the broad‐host‐range promoter *Pm* . Appl Environ Microbiol 77: 2648–2655. 21335387 10.1128/AEM.02091-10 PMC3126357

[mbt212701-bib-0056] Lan, W.S. , Lu, T.K. , Qin, Z.F. , Shi, X.J. , Wang, J.J. , Hu, Y.F. , *et al* (2014) Genetically modified microorganism *Spingomonas paucimobilis* UT26 for simultaneously degradation of methyl‐parathion and γ‐hexachlorocyclohexane. Ecotoxicology 23: 840–850. 24648032 10.1007/s10646-014-1224-8

[mbt212701-bib-0057] Li, G.W. (2015) How do bacteria tune translation efficiency? Curr Opin Microbiol 24: 66–71. 25636133 10.1016/j.mib.2015.01.001 PMC4678177

[mbt212701-bib-0058] Liu, X. , Germaine, K.J. , Ryan, D. , and Dowling, D.N. (2010) Genetically modified Pseudomonas biosensing biodegraders to detect PCB and chlorobenzoate bioavailability and biodegradation in contaminated soils. Bioeng Bugs 1: 198–206. 21326926 10.4161/bbug.1.3.12443 PMC3026425

[mbt212701-bib-0059] de Lorenzo, V. , Fernández, S. , Herrero, M. , Jakubzik, U. , and Timmis, K.N. (1993) Engineering of alkyl‐ and haloaromatic‐responsive gene expression with mini‐transposons containing regulated promoters of biodegradative pathways of Pseudomonas. Gene 130: 41–46. 8393826 10.1016/0378-1119(93)90344-3

[mbt212701-bib-0062] Maleki, S. , Almaas, E. , Zotchev, S.B. , Valla, S. , and Ertesvåg, H. (2016) Alginate biosynthesis factories in *Pseudomonas fluorescens*: localization and correlation with alginate production level. Appl Environ Microbiol 82: 1227–1236. 10.1128/AEM.03114-15 PMC4751860 26655760

[mbt212701-bib-0063] Manzanera, M. , Marqués, S. , and Ramos, J.L. (2000) Mutational analysis of the highly conserved C‐terminal residues of the XylS protein, a member of the AraC family of transcriptional regulators. FEBS Lett 476: 312–317. 10913634 10.1016/s0014-5793(00)01749-x

[mbt212701-bib-0064] Marqués, S. , Manzanera, M. , González‐Pérez, M.‐M. , Gallegos, M.‐T. , and Ramos, J.L. (1999) The XylS‐dependent *Pm* promoter is transcribed *in vivo* by RNA polymerase with σ32 or σ38 depending on the growth phase. Mol Microbiol 31: 1105–1113. 10096078 10.1046/j.1365-2958.1999.01249.x

[mbt212701-bib-0065] Martin, R.G. , and Rosner, J.L. (2001) The AraC transcriptional activators. Curr Opin Microbiol 4: 132–137. 11282467 10.1016/s1369-5274(00)00178-8

[mbt212701-bib-0066] Martínez, V. , García, P. , García, J.L. , and Prieto, M.A. (2011) Controlled autolysis facilitates the polyhydroxyalkanoate recovery in *Pseudomonas putida* KT2440. Microb Biotechnol 4: 533–547. 21418544 10.1111/j.1751-7915.2011.00257.x PMC3815265

[mbt212701-bib-0067] Martínez‐García, E. , and de Lorenzo, V. (2011) Engineering multiple genomic deletions in Gram‐negative bacteria: analysis of the multi‐resistant antibiotic profile of *Pseudomonas putida* KT2440. Environ Microbiol 13: 2702–2716. 21883790 10.1111/j.1462-2920.2011.02538.x

[mbt212701-bib-0068] Martínez‐García, E. , Nikel, P.I. , Aparicio, T. , and de Lorenzo, V. (2014) *Pseudomonas* 2.0: genetic upgrading of *P. putida* KT2440 as an enhanced host for heterologous gene expression. Microb Cell Fact 13: 159. 25384394 10.1186/s12934-014-0159-3 PMC4230525

[mbt212701-bib-0069] Medina, C. , Camacho, E.M. , Flores, A. , Mesa‐Pereira, B. , and Santero, E. (2011) Improved expression systems for regulated expression in *Salmonella* infecting eukaryotic cells. PLoS ONE 6: e23055. 21829692 10.1371/journal.pone.0023055 PMC3148252

[mbt212701-bib-0070] Mermod, N. , Ramos, J.L. , Lehrbach, P.R. , and Timmis, K.N. (1986) Vector for regulated expression of cloned genes in a wide range of gram‐negative bacteria. J Bacteriol 167: 447–454. 3525513 10.1128/jb.167.2.447-454.1986 PMC212908

[mbt212701-bib-0071] Mesa‐Pereira, B. , Medina, C. , Camacho, E.M. , Flores, A. , and Santero, E. (2013) Novel tools to analyze the function of *Salmonella* effectors show that svpb ectopic expression induces cell cycle arrest in tumor cells. PLoS ONE 8: e78458. 24205236 10.1371/journal.pone.0078458 PMC3804527

[mbt212701-bib-0072] Mesa‐Pereira, B. , Medina, C. , Camacho, E.M. , Flores, A. , and Santero, E. (2015) Improved cytotoxic effects of Salmonella‐producing cytosine deaminase in tumour cells. Microb Biotechnol 8: 169–176. 25227763 10.1111/1751-7915.12153 PMC4321383

[mbt212701-bib-0073] Michan, C. , Zhou, L. , Gallegos, M.T. , Timmis, K.N. , and Ramos, J.L. (1992a) Identification of critical amino‐terminal regions of XylS. The positive regulator encoded by the TOL plasmid. J Biol Chem 267: 22897–22901. 1429638

[mbt212701-bib-0074] Michan, C. , Kessler, B. , De Lorenzo, V. , Timmis, K.N. , and Ramos, J.L. (1992b) XylS domain interactions can be deduced from intra allelic dominance in double mutants of *Pseudomonas putida* . Mol Gen Genet 235: 406–412. 1465113 10.1007/BF00279387

[mbt212701-bib-0075] Nag, P. , and Pal, S. (2013) Fe protein over‐expression can enhance the nitrogenase activity of *Azotobacter vinelandii* . J Basic Microbiol 53: 156–162. 22581696 10.1002/jobm.201100334

[mbt212701-bib-0076] Netzer, R. , Stafsnes, M.H. , Andreassen, T. , Goksøyr, A. , Bruheim, P. , and Brautaset, T. (2010) Biosynthetic pathway for γ‐cyclic sarcinaxanthin in *Micrococcus luteus*: heterologous expression and evidence for diverse and multiple catalytic functions of C(50) carotenoid cyclases. J Bacteriol 192: 5688–5699. 20802040 10.1128/JB.00724-10 PMC2953688

[mbt212701-bib-0077] Nielsen, A.T. , Tolker‐Nielsen, T. , Barken, K.B. , and Molin, S. (2000) Role of commensal relationships on the spatial structure of a surface‐attached microbial consortium. Environ Microbiol 2: 59–68. 11243263 10.1046/j.1462-2920.2000.00084.x

[mbt212701-bib-0078] Nikel, P.I. , and de Lorenzo, V. (2013) Engineering an anaerobic metabolic regime in *Pseudomonas putida* KT2440 for the anoxic biodegradation of 1,3‐dichloroprop‐1‐ene. Metab Eng 15: 98–112. 23149123 10.1016/j.ymben.2012.09.006

[mbt212701-bib-0079] Ortiz‐Román, L. , Riquelme‐Neira, R. , Vidal, R. , and Oñate, A. (2014) Roles of genomic island 3 (GI‐3) BAB1_0267 and BAB1_0270 open reading frames (ORFs) in the virulence of *Brucella abortus* 2308. Vet Microbiol 172, 279–284. 24928771 10.1016/j.vetmic.2014.05.005

[mbt212701-bib-0080] Perlova, O. , Fu, J. , Kuhlmann, S. , Krug, D. , Stewart, A.F. , Zhang, Y. , and Müller, R. (2006) Reconstitution of the myxothiazol biosynthetic gene cluster by Red/ET recombination and heterologous expression in *Myxococcus xanthus* . Appl Environ Microbiol 72: 7485–7494. 16997979 10.1128/AEM.01503-06 PMC1694261

[mbt212701-bib-0081] Plesiat, P. , Aires, J.R. , Godard, C. , and Köhler, T. (1997) Use of steroids to monitor alterations in the outer membrane of *Pseudomonas aeruginosa* . J Bacteriol 179: 7004–7010. 9371446 10.1128/jb.179.22.7004-7010.1997 PMC179640

[mbt212701-bib-0082] Prieto, M.A. , Kellerhals, M.B. , Bozzato, G.B. , Radnovic, D. , Witholt, B. , and Kessler, B. (1999) Engineering of Stable recombinant bacteria for production of chiral medium‐chain‐length poly‐3‐hydroxyalkanoates. Appl Environ Microbiol 65: 3265–3271. 10427005 10.1128/aem.65.8.3265-3271.1999 PMC91490

[mbt212701-bib-0083] Purvanov, V. , and Fetzner, S. (2005) Replacement of active‐site residues of quinoline 2‐oxidoreductase involved in substrate recognition and specificity. Curr Microbiol 50: 217–222. 15902470 10.1007/s00284-004-4452-y

[mbt212701-bib-0084] Ramey, K. , Eko, F.O. , Thompson, W.E. , Armah, H. , Igietseme, J.U. , and Stiles, J.K. (2009) Immunolocalization and challenge studies using a recombinant *Vibrio cholerae* ghost expressing *Trypanosoma brucei* Ca^2+^ ATPase (TBCA2) antigen. Am J Trop Med Hyg 81: 407–415. 19706905 PMC2786262

[mbt212701-bib-0085] Ramos, J.L. , Stolz, A. , Reineke, W. , and Timmis, K.N. (1986) Altered effector specificities in regulators of gene expression: TOL plasmid xylS mutants and their use to engineer expansion of the range of aromatics degraded by bacteria. Proc Natl Acad Sci USA 83: 8467–8471. 3022293 10.1073/pnas.83.22.8467 PMC386951

[mbt212701-bib-0086] Ramos, J.L. , González‐Carrero, M. , and Timmis, K.N. (1988) Broad‐host range expression vectors containing manipulated meta‐cleavage pathway regulatory elements of the TOL plasmid. FEBS Lett 226: 241–246. 3123271 10.1016/0014-5793(88)81431-5

[mbt212701-bib-0087] Ramos, J.L. , Michan, C. , Rojo, F. , Dwyer, D. , and Timmis, K. (1990a) Signal‐regulator interactions. Genetic analysis of the effector binding site of *xylS*, the benzoate‐activated positive regulator of *Pseudomonas* TOL plasmid *meta‐*cleavage pathway operon. J Mol Biol 211: 373–382. 2407853 10.1016/0022-2836(90)90358-S

[mbt212701-bib-0088] Ramos, J.L. , Rojo, F. , Zhou, L. , and Timmis, K.N. (1990b) A family of positive regulators related to the *Pseudomonas putida* TOL plasmid XylS and the *Escherichia coli* AraC activators. Nucleic Acids Res 18: 2149–2152. 2186376 10.1093/nar/18.8.2149 PMC330695

[mbt212701-bib-0089] Ramos, J.L. , Marqués, S. , and Timmis, K.N. (1997) Transcriptional control of the *Pseudomonas* TOL plasmid catabolite operons is achieved through an interplay of host factors and plasmid‐encoded regulators. Annu Rev Microbiol 51: 341–373. 9343354 10.1146/annurev.micro.51.1.341

[mbt212701-bib-0090] Rosano, G.L. , and Ceccarelli, E.A. (2014) Recombinant protein expression in *Escherichia coli*: advances and challenges. Front Microbiol 5: 172. 24860555 10.3389/fmicb.2014.00172 PMC4029002

[mbt212701-bib-0091] Royo, J.L. , Moreno‐Ruiz, E. , Cebolla, A. , and Santero, E. (2005a) Stable long‐term indigo production by overexpression of dioxygenase genes using a chromosomal integrated cascade expression circuit. J Biotech 116: 113–124. 10.1016/j.jbiotec.2004.10.006 15664075

[mbt212701-bib-0192] Royo, J.L. , Manyani, H. , Cebolla, A. , and Santero, E. (2005b) A new generation of vectors with increased induction ratios by overimposing a second regulatory level by attenuation. Nucleic Acids Res 33: e169. 16260471 10.1093/nar/gni168 PMC1275594

[mbt212701-bib-0092] Royo, J.L. , Becker, P.D. , Camacho, E.M. , Cebolla, A. , Link, C. , Santero, E. , and Guzman, C.A. (2007) *In vivo* gene regulation in Salmonella spp. by a salicylate‐dependent control circuit. Nat Methods 4: 937–942. 17922017 10.1038/nmeth1107

[mbt212701-bib-0093] Ruíz, R. , and Ramos, J.L. (2001) Residues 137 and 153 of XylS influence contacts with the C‐terminal end of the RNA polymerase alpha subunit. Biochem Biophys Res Commun 287: 519–521. 11554759 10.1006/bbrc.2001.5615

[mbt212701-bib-0094] Ruíz, R. , and Ramos, J.L. (2002) Residues 137 and 153 at the N terminus of the XylS protein influence the effector profile of this transcriptional regulator and the s factor used by RNA polymerase to stimulate transcription from its cognate promoter. J Biol Chem 277: 7282–7286. 11751934 10.1074/jbc.M110226200

[mbt212701-bib-0095] Ruíz, R. , Marqués, S. , and Ramos, J.L. (2003) Leucines 193 and 194 at the N‐Terminal domain of the XylS protein, the positive transcriptional regulator of the TOL *meta*‐cleavage pathway, are involved in dimerization. J Bacteriology 185: 3036–3041. 10.1128/JB.185.10.3036-3041.2003 PMC154087 12730162

[mbt212701-bib-0096] Santiago, A.E. , Yan, M.B. , Tran, M. , Wright, N. , Luzader, D.H. , Kendall, M.M. , *et al* (2016) A large family of anti‐activators accompanying XylS/AraC family regulatory proteins. Mol Microbiol 101: 314–332. 27038276 10.1111/mmi.13392 PMC4983702

[mbt212701-bib-0097] Sasoh, M. , Masai, E. , Ishibashi, S. , Hara, H. , Kamimura, N. , Miyauchi, K. , and Fukuda, M. (2006) Characterization of the terephthalate degradation genes of *Comamonas* sp. strain E6. Appl Environ Microbiol 72: 1825–1832. 16517628 10.1128/AEM.72.3.1825-1832.2006 PMC1393238

[mbt212701-bib-0098] Schleif, R. (2003) AraC protein: a love‐hate relationship. BioEssays 25: 274–282. 12596232 10.1002/bies.10237

[mbt212701-bib-0099] Schmutzler, K. , Schmid, A. , and Buehler, K. (2015) A three‐step method for analysing bacterial biofilm formation under continuous medium flow. Appl Microbiol Biotechnol 99: 6035–6047. 25936379 10.1007/s00253-015-6628-8

[mbt212701-bib-0102] Silva‐Rocha, R. , Jong, H. , Tamames, J. , and de Lorenzo, V. (2011) The logic layout of the TOL network of *Pseudomonas putida* pWW0 plasmid stems from a metabolic amplifier motif (MAM) that optimizes biodegradation of *m*‐xylene. BMC Syst Biol 5: 191. 22078029 10.1186/1752-0509-5-191 PMC3253710

[mbt212701-bib-0103] Silva‐Rocha, R. , Martínez‐García, E. , Calles, B. , Chavarría, M. , Arce‐Rodríguez, A. , de las Heras, A. , *et al* (2013) The Standard European Vector Architecture (SEVA): a coherent platform for the analysis and deployment of complex prokaryotic phenotypes. Nucleic Acids Res, 41 (Database issue): D666–D675. 23180763 10.1093/nar/gks1119 PMC3531073

[mbt212701-bib-0204] Silva‐Rocha, R. , and de Lorenzo, V. (2014) The pWW0 plasmid imposes a stochastic expression regime to the chromosomal ortho pathway for benzoate metabolism in Pseudomonas putida. FEMS Microbiol Lett 356: 176–183. 25848660 10.1111/1574-6968.12400

[mbt212701-bib-0104] Sletta, H. , Nedal, A. , Aune, T.E. , Hellebust, H. , Hakvåg, S. , Aune, R. , *et al* (2004) Broad‐host‐range plasmid pJB658 can be used for industrial‐level production of a secreted host‐toxic single‐chain antibody fragment in *Escherichia coli* . Appl Environ Microbiol 70: 7033–7039. 15574897 10.1128/AEM.70.12.7033-7039.2004 PMC535149

[mbt212701-bib-0105] Sletta, H. , Tøndervik, A. , Hakvåg, S. , Aune, T.E. , Nedal, A. , Aune, R. , *et al* (2007) The presence of N‐terminal secretion signal sequences leads to strong stimulation of the total expression levels of three tested medically important proteins during high‐cell‐density cultivations of *Escherichia coli* . Appl Environ Microbiol 73: 906–912. 17142370 10.1128/AEM.01804-06 PMC1800768

[mbt212701-bib-0106] Steigedal, M. , Sletta, H. , Moreno, S. , Mærk, M. , Christensen, B.E. , Bjerkan, T. , *et al* (2008) The *Azotobacter vinelandii* AlgE mannuronan C‐5‐epimerase family is essential for the *in vivo* control of alginate monomer composition and for functional cyst formation. Environ Microbiol 10: 1760–1770. 18373676 10.1111/j.1462-2920.2008.01597.x

[mbt212701-bib-0107] Studier, F.W. , and Moffatt, B.A. (1986) Use of bacteriophage T7 RNA polymerase to direct selective high‐level expression of cloned genes. J Mol Biol 189: 113–130. 3537305 10.1016/0022-2836(86)90385-2

[mbt212701-bib-0108] Tegel, H. , Ottosson, J. , and Hober, S. (2011) Enhancing the protein production levels in *Escherichia coli* with a strong promoter. FEBS J 278: 729–739. 21205203 10.1111/j.1742-4658.2010.07991.x

[mbt212701-bib-0109] Terpe, K. (2006) Overview of bacterial expression systems for heterologous protein production: from molecular and biochemical fundamentals to commercial systems. Appl Microbiol Biotechnol 72: 211–222. 16791589 10.1007/s00253-006-0465-8

[mbt212701-bib-0111] Tobes, R. , and Ramos, J.L. (2002) AraC‐XylS database: a family of positive transcriptional factors in bacteria. Nucl Acids Res 30: 318–321. 11752325 10.1093/nar/30.1.318 PMC99111

[mbt212701-bib-0112] Tøndervik, A. , Balzer, S. , Haugen, T. , Sletta, H. , Rode, M. , Lindmo, K. , *et al* (2013) High production of recombinant Norwegian salmonid alphavirus E1 and E2 proteins in *Escherichia coli* by fusion to secretion signal sequences and removal of hydrophobic domains. Biotechnol Bioproc E 18: 742–750.

[mbt212701-bib-0113] Valls, M. , Atrian, S. , de Lorenzo, V. , and Fernández, L.A. (2000) Engineering a mouse metallothionein on the cell surface of *Ralstonia eutropha* CH34 for immobilization of heavy metals in soil. Nat Biotechnol 18: 661–665. 10835606 10.1038/76516

[mbt212701-bib-0114] Volmer, J. , Neumann, C. , Bühler, B. , and Schmid, A. (2014) Engineering of *Pseudomonas taiwanensis* VLB120 for Constitutive Solvent Tolerance and Increased Specific Styrene Epoxidation Activity. Appl Environ Microbiol 80: 6539–6548. 25128338 10.1128/AEM.01940-14 PMC4178659

[mbt212701-bib-0115] Wenzel, S.C. , Gross, F. , Zhang, Y. , Fu, J. , Stewart, A.F. , and Müller, R. (2005) Heterologous expression of a myxobacterial natural products assembly Line in pseudomonads via Red/ET recombineering. Chem Biol 12: 349–356. 15797219 10.1016/j.chembiol.2004.12.012

[mbt212701-bib-0116] Winther‐Larsen, H.C. , Josefsen, K.D. , Brautaset, T. , and Valla, S. (2000a) Parameters affecting gene expression from the *Pm* promoter in gram‐negative bacteria. Met Eng 2: 79–91. 10.1006/mben.1999.0142 10935724

[mbt212701-bib-0117] Winther‐Larsen, H.C. , Blatny, J.M. , Valand, B. , Brautaset, T. , and Valla, S. (2000b) *Pm* promoter expression mutants and their use in broad‐host‐range RK2 plasmid vectors. Metab Eng 2: 92–103. 10935725 10.1006/mben.1999.0143

[mbt212701-bib-0119] Worsey, M.J. , and Williams, P.A. (1975) Metabolism of toluene and xylenes by *Pseudomonas putida* (arvilla) mt‐2: evidence for a new function of the TOL plasmid. J Bacteriol 124: 7–13. 1176436 10.1128/jb.124.1.7-13.1975 PMC235858

[mbt212701-bib-0120] Yang, Y. , Yuan, S. , Chen, T. , Ma, P. , Shang, G. , and Dai, Y. (2009) Cloning, heterologous expression, and functional characterization of the nicotinate dehydrogenase gene from *Pseudomonas putida* KT2440. Biodegradation 20: 541–549. 19118407 10.1007/s10532-008-9243-x

[mbt212701-bib-0121] Yao, J. , and Lambowitz, A.M. (2007) Gene targeting in gram‐negative bacteria by use of a mobile group II intron (“Targetron”) expressed from a broad‐host‐range vector. Appl Environ Microbiol 73: 2735–2743. 17322321 10.1128/AEM.02829-06 PMC1855620

[mbt212701-bib-0122] Zhang, L. , and Mah, T.‐F. (2008) Involvement of a novel efflux system in biofilm‐specific resistance to antibiotics. J Bacteriol 190: 4447–4452. 18469108 10.1128/JB.01655-07 PMC2446775

[mbt212701-bib-0123] Zhang, L. , Hinz, A.J. , Nadeau, J.‐P. , and Mah, T.‐F. (2011) *Pseudomonas aeruginosa tssC1* links type VI secretion and biofilm‐specific antibiotic resistance. J Bacteriol 193: 5510–5513. 21784934 10.1128/JB.00268-11 PMC3187457

[mbt212701-bib-0124] Zhang, L. , Fritsch, M. , Hammond, L. , Landreville, R. , Slatculescu, C. , Colavita, A. , and Mah, T.‐F. (2013) Identification of genes involved in *Pseudomonas aeruginosa* biofilm‐specific resistance to antibiotics. PLoS ONE 8: e61625. 23637868 10.1371/journal.pone.0061625 PMC3634840

[mbt212701-bib-0125] Zhou, L.M. , Timmis, K.N. , and Ramos, J.L. (1990) Mutations leading to constitutive expression from the TOL plasmid *meta*‐cleavage pathway operon are located at the C‐terminal end of the positive regulator protein XylS. J Bacteriol 172: 3707–3710. 2193914 10.1128/jb.172.7.3707-3710.1990 PMC213347

[mbt212701-bib-0126] Zhuang, F. , Karberg, M. , Perutka, J. , and Lambowitz, A.M. (2009) EcI5, a group IIB intron with high retrohoming frequency: DNA target site recognition and use in gene targeting. RNA 15: 432–449. 19155322 10.1261/rna.1378909 PMC2657007

[mbt212701-bib-0127] Zwick, F. , Lale, R. , and Valla, S. (2012) Strong stimulation of recombinant protein production in *Escherichia coli* by combining stimulatory control elements in an expression cassette. Microb Cell Fact 11: 133. 23031552 10.1186/1475-2859-11-133 PMC3526546

[mbt212701-bib-0128] Zwick, F. , Lale, R. , and Valla, S. (2013) Regulation of the expression level of transcription factor XylS reveals new functional insight into its induction mechanism at the Pm promoter. BMC Microbiol 13: 262. 24252441 10.1186/1471-2180-13-262 PMC4225500

